# A m^6^Avalue predictive of prostate cancer stemness, tumor immune landscape and immunotherapy response

**DOI:** 10.1093/narcan/zcac010

**Published:** 2022-03-25

**Authors:** Cheng Zou, Qinju He, Yuqing Feng, Mengjie Chen, Dingxiao Zhang

**Affiliations:** School of Biomedical Sciences, Hunan University, Changsha 410082, China; College of Animal Science and Veterinary Medicine, Huazhong Agricultural University, Wuhan 430070, China; School of Biomedical Sciences, Hunan University, Changsha 410082, China; School of Biomedical Sciences, Hunan University, Changsha 410082, China; School of Biomedical Sciences, Hunan University, Changsha 410082, China; School of Biomedical Sciences, Hunan University, Changsha 410082, China

## Abstract

The molecular mechanisms underpinning prostate cancer (PCa) progression are incompletely understood, and precise stratification of aggressive primary PCa (pri-PCa) from indolent ones poses a major clinical challenge. Here, we comprehensively dissect, genomically and transcriptomically, the m^6^A (*N*^6^-methyladenosine) pathway as a whole in PCa. Expression, but not the genomic alteration, repertoire of the full set of 24 m^6^A regulators at the population level successfully stratifies pri-PCa into three m^6^A clusters with distinct molecular and clinical features. These three m^6^A modification patterns closely correlate with androgen receptor signaling, stemness, proliferation and tumor immunogenicity of cancer cells, and stroma activity and immune landscape of tumor microenvironment (TME). We observe a discrepancy between a potentially higher neoantigen production and a deficiency in antigen presentation processes in aggressive PCa, offering insights into the failure of immunotherapy. Identification of PCa-specific m^6^A phenotype-associated genes provides a basis for construction of m^6^Avalue to measure m^6^A methylation patterns in individual patients. Tumors with lower m^6^Avalue are relatively indolent with abundant immune cell infiltration and stroma activity. Interestingly, m^6^Avalue separates PCa TME into fibrotic and nonfibrotic phenotypes (instead of previously reported immune-proficient or -desert phenotypes in other cancer types). Significantly, m^6^Avalue can be used to predict drug response and clinical immunotherapy efficacy in both castration-resistant PCa and other cancer types. Therefore, our study establishes m^6^A methylation modification pattern as a determinant in PCa progression via impacting cancer cell aggressiveness and TME remodeling.

## Graphical Abstract

**Graphical Abstract:**
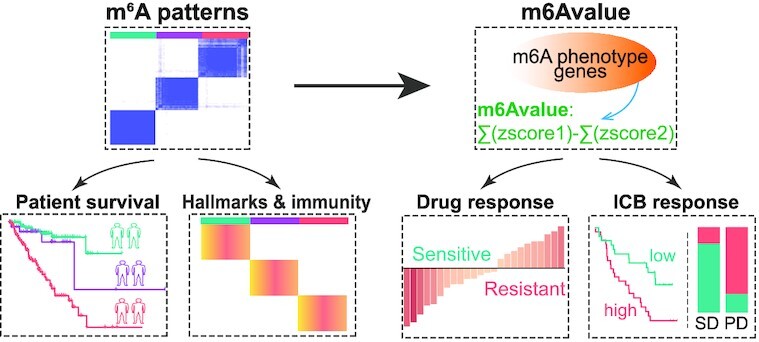
A m6Avalue predictive of prostate cancer aggressiveness and immunity.

## INTRODUCTION

Human prostate cancer (PCa) is the second most frequent diagnosed malignancy in men worldwide, counting 1 414 259 new cases and causing 375 304 deaths (3.8% of all deaths caused by cancer in men) in 2020 (1). For years, PCa has ranked the first and the second cancer type for incidence and mortality in the United States, respectively (2). The prostate is an exocrine gland containing androgen receptor negative (AR^−^) basal and androgen-sensitive AR^+^ luminal epithelial cells, together with rare neuroendocrine (NE) cells (3,4). PCa develops over a long period of time from normal prostate to prostatic intraepithelial neoplasia, then to early- and late-stage primary PCa (pri-PCa), and finally to metastatic PCa with or without treatment (5). Histologically, PCa presents as adenocarcinoma with a predominant luminal phenotype. Clinically, most pri-PCa are diagnosed as low to intermediate grade [i.e. Gleason grade (GS) ≤ 7], relatively indolent and treated by radical prostatectomy and/or radiation with a good prognosis (6). In contrast, locally advanced (GS ≥ 9) and metastatic PCa are mainly treated with androgen deprivation therapy (ADT, such as luteinizing hormone-releasing hormone agonists/antagonists) to block testicular androgen synthesis (7), but most cases will eventually fail ADT, recur and result in a lethal disease termed castration-resistant PCa (CRPC) (4,5). CRPCs are currently treated with anti-androgens such as enzalutamide (Enza) that interfere with AR functions, but unfortunately patients will succumb to recurrence in 4–5 months (6). Cancer stemness, manifested by stem cell (SC)-like properties of cancer cells, has been widely appreciated as a key determinant in tumor progression and therapy resistance (8). We have recently shown that CRPCs are relatively undifferentiated and, molecularly, basal/stem-like (3). In support, knockout of the full-length AR in androgen-sensitive LNCaP cells elicits an SC-like phenotype with enhanced proliferation and CRPC-regenerating ability under castration conditions (9).

The mechanisms underlying PCa progression, especially treatment resistance and subsequent maintenance of CRPC, are incompletely understood (10). In the past decade, owing to the global efforts of applying high-throughput next-generation sequencing technology in clinic [typified by The Cancer Genome Atlas (TCGA) program], we have now reached a consensus that genomic alteration, transcriptomic abnormality and epigenetic dysregulation all play pivotal roles in prostate tumorigenesis (3,6,9,11–17). We have recently shown that the copy number variations (CNVs) and mRNA expression disturbance of splicing regulatory genes (SRGs) jointly contribute to RNA splicing dysregulation seen in aggressive PCa, which offers a novel therapeutic vulnerability by targeting spliceosome (6). In another effort to dissect the AR heterogeneity and distinct treatment responses, we found that an SC transcriptional program driven by BCL-2 is operating in AR^−/low^ CRPCs and our proof-of-concept studies have validated a combinatorial therapy (BCL-2 inhibitor plus Enza) as an efficient therapeutic regimen for both AR^+^ and AR^−/low^ CRPCs (9). Moreover, in addition to CRPC emergence, distinguishing the many indolent pri-PCa from the minority of lethal ones (otherwise leading to overtreatment) represents another major clinical challenge (18). Based on gene expression profiles within low-GS prostate tumors, a 19-gene signature was previously identified to distinguish indolent versus aggressive subgroups (19). In light of splicing regulation and correlation with worse clinical outcome, we have recently developed a 13-SRG signature to separate aggressive pri-PCa from indolent ones (6). Notably, despite the research progress, PCa still causes a significant mortality. Together with the fact of interpatient heterogeneity of PCa at both molecular and clinical levels, more studies are needed to interrogate PCa evolution at distinct angles.

Despite the multilayers of regulatory mechanisms in PCa etiology, they all converge, eventually, on gene expression regulation at versatile levels, as gene expression is the fundamental determinant of cellular phenotypes (3). *N*^6^-Methyladenosine (m^6^A), the most abundant form of internal modifications in eukaryote RNA, post-transcriptionally modulates gene expression by impacting RNA biology [e.g. stability, subcellular localization, transportation, translation and alternative splicing (AS)], and thus functions in a spectrum of important bioprocesses (20). Expectedly, emerging evidence has implicated m^6^A modification in tumorigenesis of diverse organ systems (21). Analogous to epigenetic DNA methylation, m^6^A is a dynamic RNA modification and is controlled by the methyltransferase ‘writer’ complex, the demethylase ‘erasers’ and ‘reader’ proteins (22,23). In PCa, a few reports, on an individual gene basis, have highlighted the significance of m^6^A methylation in tumorigenesis. For example, *METTL3* has been shown to be both highly expressed in PCa tissues and essential for proliferation and metastasis in multiple PCa cell lines (24–26). Knocking down of *YTHDF2* suppresses proliferation and migration of PCa cells by globally elevating m^6^A levels (27). However, given that the cellular m^6^A homeostasis is established by an integrated network of m^6^A regulators (consisting of all writers/erasers/readers within a cell), a comprehensive study that considers the m^6^A pathway as a whole has not been performed in PCa. More interestingly, besides the roles of m^6^A in cancer cells *per se*, m^6^A has been recognized to regulate the fate of immune cells and thus reshape the immune landscape of tumor microenvironment (TME) (28–31). Evidence has unraveled several m^6^A regulators as key molecules modulating tumor immunity and responses to immunotherapies typified by immune checkpoint blockades (ICBs) (32–35). PCa is immunologically ‘cold’ due to immunosuppressive TME and poor immune infiltration (36), but clinical trials have observed, encouragingly, that a small proportion of patients did exhibit beneficial responses to ICB (such as anti-PD-1/PD-L1/CTLA-4) (37). Experimentally, studies have shown that PCa can be induced to be amenable for ICB by induction of an effective antitumor response via TME remodeling (38,39). In particular, a tight association between m^6^A modification patterns and TME diversity and complexity has been suggested in gastric (40) and colon (41) cancers, but whether the m^6^A pathway plays a role in PCa immunity remains elusive.

**Figure 1.**
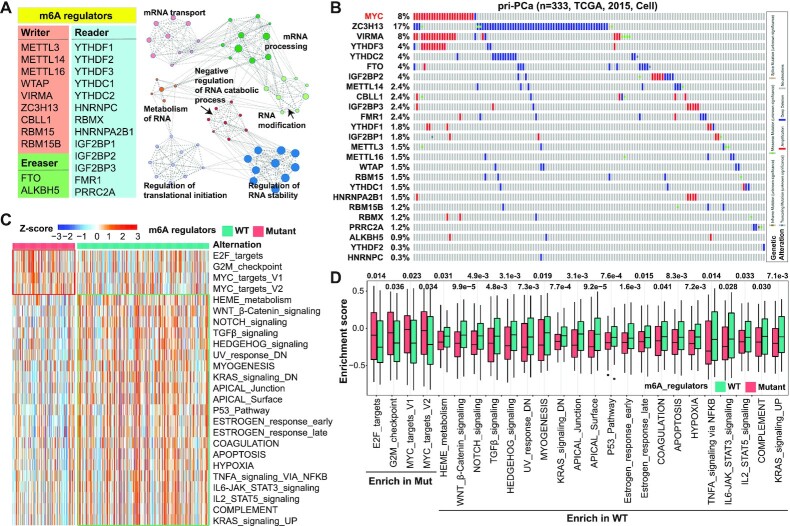
Mutational landscape of m^6^A regulators in human pri-PCa. (**A**) List of the full set of currently known 24 m^6^A regulators (left) and functional network of the top 7 biological categories enriched in these regulators by Metascape analysis (right). (**B**) A comprehensive survey of genomic alterations in 24 m^6^A regulators in the curated TCGA clinical cohort in cBioPortal. Frequently amplified *MYC* (colored in red) is included as a reference gene. Each bar represents the alteration status of an individual gene for a single patient and the percentage of alterations for each gene in the cohort is provided. Heatmap presentation (**C**) and boxplot quantification (**D**) of enrichment of the representative cancer hallmarks from Molecular Signatures Database (MSigDB) by gene set variation analysis (GSVA) in pri-PCa with (mutant group) or without [wild-type (WT) group] genomic alternations in m^6^A regulators. Within the plots, the center lines represent median values, box edges are 75th and 25th percentiles, and dots denote the outliers. Significance was calculated by the Wilcoxon test.

Here, we focus on the m^6^A pathway as a whole and provide a comprehensive characterization of different m^6^A methylation patterns during PCa evolution. We report that m^6^A dysregulation, caused by a global upregulation of many writer and reader genes and downregulation of eraser genes, plays an oncogenic role in PCa. The expression repertoire of m^6^A regulators clearly classifies PCa into three clusters with distinct molecular and clinical features. We also systematically correlate the m^6^A modification patterns with AR signaling, stemness, tumor immunogenicity and immune landscape in PCa. To facilitate our findings toward a potential clinical usage, we establish m^6^Avalue, a scoring signature based on m^6^A phenotype-associated genes, to quantify the m^6^A modification pattern in individual PCa patients. We demonstrate that m^6^Avalue associates positively with a nonfibrotic TME phenotype that augments aggressiveness and can be used to predict responses to both small-molecule inhibitors and immunotherapies in PCa and other cancer types.

## MATERIALS AND METHODS

### Data collection and bioinformatic preprocessing

In this study, we utilized a total of four large PCa cohorts, including two TCGA [both curated (11) and noncurated pan-cancer] and two Gene Expression Omnibus (GEO) validation cohorts [GSE21034 (42) and GSE116918 (43)]. For study of immunotherapy responses, four cohorts including a CRPC (44) and three melanoma [GSE78220 (45), GSE91061 (46) and GSE100797 (47)] cohorts were used. Public gene expression and clinical information was downloaded from TCGA and NCBI GEO database. For RNA sequencing (RNA-seq) data, gene expression matrixes (read counts or FPKM) were downloaded via the R package TCGAbiolinks (48) or GEO database. The original gene expression values were transformed into transcripts per kilobase million format. For microarray data, the R package GEOquery (49) was used to download the raw data and further extract gene expression based on the platform information. Somatic mutation data were obtained from TCGA by the R package TCGAmutations (50). Also, for visualization, we analyzed the landscape of genomic alternations of 24 m^6^A regulators, together with reference genes MYC and RB1, by cBioPortal (51).

### Identification of DEGs and DSEs

For differentially expressed gene (DEG) analysis, the read count files of the curated (333 tumors) and noncurated pan-cancer TCGA-PRAD cohorts (*n* = 494) were used. First, genes with at least three reads in more than one-third of tumors were retained for further analysis. Then, the DESeq2 package (52) was applied to identified DEGs with cutoff of false discovery rate (FDR) <0.05 and fold change >2. For differentially spliced event (DSE) analysis, raw RNA-seq files were mapped to human reference genome (Homo_sapiens.GRCh38.dna.primary_assembly.fa) by STAR 2.7.3a (53), and then quantified with rMATS v4.0 (54) to identify distinct types of splicing events. DSEs with FDR < 0.1 and ΔPSI (percent of splicing inclusion) > 0.1 were considered significant events.

### Association between DEGs and m^6^A targets

In order to verify that the DEGs identified from different m^6^A modification patterns were indeed targets of the m^6^A pathway, we overlapped them with two sets of m^6^A targets. First, we downloaded the potential target genes of m^6^A regulators (human) from m6A2Target (http://m6A2target.canceromics.org/#/download) (55). Particularly, m6A2Target recorded target genes derived from a spectrum of experimental and/or bioinformatic methods. To increase the confidence of our results, potential target genes that were predicted by at least two strategies were chosen. Then, these target genes were grouped by different m^6^A regulators and a hypergeometric test was applied to analyze the overlap between DEGs and m^6^A target genes. Second, we performed m^6^A-seq in three clinical PCa samples and identified a total of 14 354 genes bearing m^6^A peaks (Supplementary Table S1). Overlapping of DEGs with this set of experimentally validated m^6^A target genes further strengthened the clinical relevance of our results.

### m^6^A-seq analysis

The m^6^A-seq analysis was performed in collaboration with LC Sciences, LLC. Briefly, total RNA was isolated from prostate tissues using TRIzol reagent (Invitrogen, Carlsbad, CA, USA), followed by two rounds of poly(A)+ RNA purification using Dynabeads Oligo (dT)25-61005 (Thermo Fisher, CA, USA). Then, the poly(A)+ RNA was sheared into 100–200-nt fragments using Magnesium RNA Fragmentation Module (NEB, USA, cat # e6150). A portion of the RNA fragments was directly used as input for regular RNA-seq, and another portion was incubated for 2 h at 4°C with anti-m^6^A antibody (No. 202003, Synaptic Systems, Germany) in IP buffer (50 mM Tris–HCl, 750 mM NaCl and 0.5% Igepal CA-630). The m^6^A–IP RNA mixture was then incubated with Dynabeads protein A/G for an additional 2 h at 4°C on a rotating wheel. After washing three times with IP buffer, the bound RNA was purified for downstream library preparation and sequencing on an illumina Novaseq™ 6000 (LC-Bio Technology Co., Ltd, Hangzhou, China). For data analysis, HISAT2 (http://daehwankimlab.github.io/hisat2) was used to map reads to the reference genome *Homo sapiens GRCh38*. The m^6^A peaks were then identified by HOMER (http://homer.ucsd.edu/homer) and macs2 (https://github.com/taoliu/MACS), and only peaks identified by two software were retained. We utilized HOMER (http://homer.ucsd.edu/homer/motif) for *de novo* and known motif finding followed by localization of the motif with respect to peak summit.

### Gene set variation analysis and functional annotation

We utilized the ‘GSVA’ package (56) to conduct GSVA enrichment analysis. The gene-set libraries of ‘h.all.v7.4.symbols.gmt’ and ‘c2.all.v7.4.symbols.gmt’ were downloaded from MSigDB. In addition, we also curated a list of previously reported and biologically relevant gene signatures (Supplementary Table S2). For example, an AR signature was previously established by assessing the expression levels of 30 genes that were previously reported as defining the pathway (57). A 109-gene signature that excluded confounding immune genes and proliferation markers has been validated in multiple cancer types to faithfully recapitulate stemness (58). The comparative enrichment score was calculated for each gene signature and *P*-value <0.05 was considered statistically significant. In parallel, we also used gene set enrichment analysis (GSEA) (59), based on the pre-ranked expression list, to annotate transcriptomic profiles. We followed the standard procedure described by GSEA user guide. The FDR for GSEA is the estimated probability that a gene set with a given NES (normalized enrichment score) represents a false-positive finding and an FDR < 0.25 is considered to be statistically significant. The Metascape (http://metascape.org) was used to annotate DEGs. Terms with *P* < 0.05, minimum count 3 and enrichment factor >1.5 (enrichment factor is the ratio between observed count and the count expected by chance) were considered significant. The STRING database was used to construct protein–protein interaction network and disconnected nodes in the network were discarded.

### Consensus clustering for 24 m^6^A regulators

We systematically investigated the full set of currently known 24 m^6^A regulators (23) and utilized unsupervised clustering to identified different m^6^A modification patterns based on their expression in a given PCa cohort. The number of clusters was determined by the consensus clustering algorithm in the ConsensusClusterPlus package (60) and a permutation of 1000 times was used to stringently increase our classification reliability.

### Cancer immunity cycle analysis and quantification of TME cell infiltration

A previous study has conceptualized the anticancer immune response as seven sequential steps: (i) release of cancer cell antigens; (ii) cancer antigen presentation; (iii) priming and activation; (iv) trafficking of immune cells to tumors; (v) infiltration of immune cells into tumors; (vi) recognition of cancer cells by T cells; and (vii) killing of cancer cells. In aggregate, these seven steps were referred to as the cancer immunity cycle (61). Based on the gene markers specific to each step, we used GSVA to estimate the activation or activity of each step. For immune cell infiltration, a previously well-established compendium of gene signatures related to 28 specific immune cells (62) was used to quantify the relative abundance of each tumor-infiltrating lymphocyte (TIL) subpopulation by GSVA. We utilized R package ESTIMATE (63) to evaluate the ImmuneScore and StromaScore in PCa TME.

### Construction of the weighted m^6^A gene signature and m^6^Avalue

**Figure 2.**
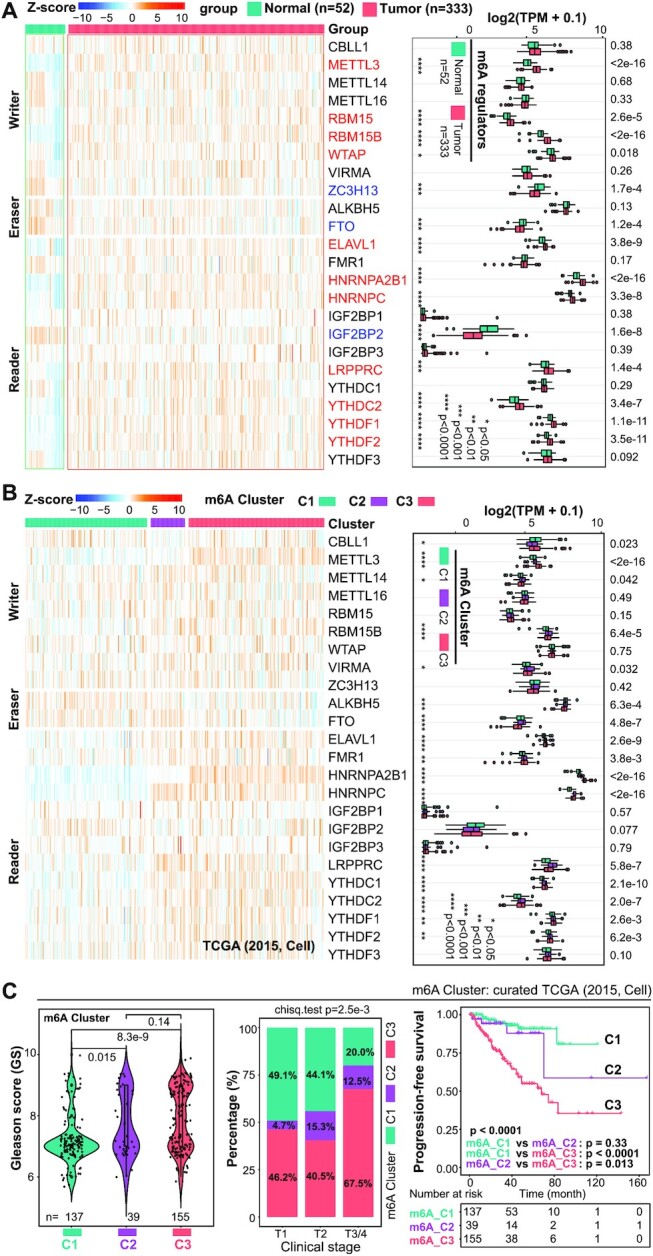
Distinct m^6^A modification patterns classify PCa into three clusters with distinct clinical features. (**A**) Heatmap presentation (left) and boxplot quantification (right) of the expression pattern of 24 m^6^A regulators between normal prostate (*n* = 52) and pri-PCa (*n* = 333) tissues. Gene expression is normalized by *Z*-score, with red and blue denoting a high and a low expression, respectively. Up- and downregulated genes are colored in red and blue, respectively (right). Within the plots, the center lines represent median values, box edges are 75th and 25th percentiles, and dots denote the outliers. Significance was calculated by the Wilcoxon test. (**B**) The expression repertoire of 24 m^6^A regulators classifies pri-PCa into three clusters (termed m^6^A_Cluster1/2/3) in the curated TCGA cohort (left), with an overall upregulation of many writer and reader genes and downregulation of eraser genes in C3 versus C1. Boxplot (right) showing difference in expression of 24 m^6^A regulators among three m^6^A_Clusters. Significance was calculated by the Kruskal–Wallis test. *P < 0.05, **P < 0.01, ***P < 0.001 and ****P < 0.0001. (**C**) Comparison of GS (left), tumor stage (middle) and patients’ survival (right) showing m^6^A clusters C1 and C3 as the least and most aggressive PCa subtypes, respectively. The proportion of patients clustered into m^6^A_C2/3 in advanced T3/4 stages (67.5% + 12.5% = 80%) is much higher than that in T1 (46.2% + 4.7% = 50.9%) and T2 (40.5% + 15.3% = 55.8%) stages (middle). Significance was calculated by the Kruskal–Wallis test (left), chi-square test (middle) and long-rank test (right), respectively. A ‘jittered’ function of ggplot2 was used for GS visualization.

Construction of the weighted m^6^A gene signature (reflecting the overall m^6^A activity or level) was calculated based on a linear *Z*-score combination of expression of 24 regulators. In particular, 4 genes (ALKBH5, FTO, ZC3H13 and IGF2BP2) downregulated in tumors versus normal tissue and other 20 genes with a trend of upregulation in tumors were considered negatively and positively weighted, respectively (Figure 2A, right).


\begin{equation*}{\mathrm{m}}6{\mathrm{A}} \; {\mathrm{gene\ }} = \mathop \sum \limits_{\mathrm{\ }}^{\mathrm{\ }} {\mathrm{zscore}}\_20\_{\mathrm{genes}} - \mathop \sum \limits_{\mathrm{\ }}^{\mathrm{\ }} {\mathrm{zscore}}\_4\_{\mathrm{genes }}.\ \end{equation*}

Construction of the m^6^Avalue was performed as follows. First, expression pattern of the 24 regulators well separated pri-PCa into three clusters (m^6^A_C1/2/3) at the population level, and 407 core DEGs were identified (via collecting the overlapping genes in comparisons of any two clusters) as m^6^A phenotype-associated genes (Supplementary Figure S3A). Then, we performed prognostic analysis for each core DEG by a univariate Cox model and 87 genes with significant prognosis (*P* < 0.05) were extracted for following analysis. Next, the m^6^Avalue was calculated based on a linear combination of expression values (*Z*-score) of the 87 genes, of which genes with hazard ratio (HR) >1 and <1 contributed positively and negatively to m^6^Avalue, respectively:


\begin{equation*} {\rm m}^6{\rm Avalue}=\sum {\rm zscore}1-\sum {\rm zscore}2 ,\end{equation*}

where zscore1 refers to expression value of genes with HR > 1 and zscore2 refers to expression value of genes with HR < 1.

### Association of m^6^Avalue with drug sensitivity

The transcriptomes of 469 cancer cell lines and the corresponding drug response information (i.e. IC_50_) of 24 anticancer drugs were downloaded from CCLE (Cancer Cell Line Encyclopedia) database (https://portals.broadinstitute.org/ccle). The m^6^Avalue for each cell line was calculated by the formula described earlier. Spearman correlation was performed to estimate the association between m^6^Avalue and drug response and a correlation with |*r*| > 0.1 and *P*-value <0.05 was considered significant.

### Statistical analysis

All statistical analysis in our study were performed by R 4.1.0. Specifically, the m^6^A regulator co-expression analysis, association between m^6^Avalue and selected signatures, cancer immunity cycle and 28 TIL subpopulations were performed by Pearson analysis (Figure 6A; Supplementary Figures S2A and S6A). Quantitative data fitting normal distribution were compared by *t*-test; otherwise, the Wilcoxon (for two groups) or Kruskal–Wallis (for more than two groups) test was used. Chi-square or Fisher’s exact test was performed to compare differences between categorical variables. Kaplan–Meier survival analysis and Cox regression model were used to analyze the prognostic value of m^6^Avalue by the packages ‘survival’ and ‘survminer’, and the long-rank test was utilized to determine the significance. The ‘survcutpoint’ function was applied to determine the optimal cutoff for m^6^Avalue with maximum rank statistic. The pROC package was used to estimate the specificity and sensitivity of the m^6^Avalue model. Heatmaps showing gene expression (Figures 2A, left, and B, left, and 5A; Supplementary Figures S1D, S2E, left, and S5B) or signature activity (Figures 1C and 4A and C–F; Supplementary Figures S1E and G, S4C–E and S5D–G) were visualized based on normalized *Z*-score values. For clinical outcome analyses of the curated TCGA-PRAD cohort, two samples (TCGA-HC-7740 and TCGA-HC-8265) were excluded due to duplicate existing for each sample in the cohort (Figures 2C and 5B–E; Supplementary Figures S1C and S2C).

## RESULTS

### Genomic alternations of m^6^A regulators unclearly classify PCa

A total of currently known 24 m^6^A regulators (9 writers, 2 erasers and 13 readers) (23) were investigated (Figure 1A). Functional annotation of these 24 genes showed that, expectedly, they exclusively regulated cellular processes concentrating on RNA biology (e.g. RNA modification, stability and translation) (Figure 1A). To explore the molecular mechanisms underpinning the m^6^A signaling dysregulation in PCa, we first examined genetic alterations in these regulators in pri-PCa. We mainly utilized the previously published TCGA-PRAD cohort (11) throughout the study, as it was curated. Among the 333 patients in this largest curated pri-PCa cohort (11), CNV represented the main alteration form (Figure 1B), with ZC3H13, YTHDC2 and FTO being the most deleted genes and VIRMA and YTHDF3 being the most amplified genes. Interestingly, the top deleted and amplified genes often co-occurred with the deletion of tumor suppressor genes and amplification of oncogenes, respectively. For example, ZC3H13 and RB1 were colocalized and codeleted on Chr13q (*P* < 0.001, one-sided Fisher’s exact test) (Supplementary Figure S1A). On the other hand, VIRMA and YTHDF3 were co-amplified with MYC on 8q (*P* < 0.001) (Figure 1B). Globally, we observed a weak mutational co-occurrence relationship among 24 regulators (Supplementary Figure S1B). Importantly, interrogation of the TCGA pan-cancer cohort (*n* = 494) generated a similar mutational landscape (Supplementary Figure S1A). In aggregate, our data indicate that, albeit a low alteration frequency at individual gene level (≤3 genes mutated at a rate of >5% in indicated cohorts), m^6^A signaling, collectively, represents a frequently mutated pathway in pri-PCa, as 40% and 33% of patients recorded in the curated TCGA cohort (11) and TCGA pan-cancer cohort, respectively, harbor at least one mutation of one m^6^A regulator (Figure 1B; Supplementary Figure S1A).

Somatic alternations are major drivers of cancer development (64). Classification of patients with or without mutations in these m^6^A regulators showed that the mutant group displayed a nonsignificant trend of worse survival outcome, but significantly higher GS, over the WT group (Supplementary Figure S1C), suggesting a potential pro-oncogenic role for the deregulated m^6^A pathway. Further, GSVA unraveled that many cancer hallmarks were dysregulated between these two groups (Figure 1C), with a noticeable pattern of an enrichment of proliferation pathways (e.g. E2F targets, G2M checkpoint and MYC targets) in the mutant group. However, pathways of TGFβ, p53 and apoptosis were more activated in the WT group (Figure 1D), again indicating that tumors with aberrant m^6^A signaling might be more aggressive. To further characterize the molecular difference between mutant versus WT groups, we identified a total of 132 DEGs (37 upregulated and 95 downregulated) (Supplementary Figure S1D). Gene Ontology (GO) analysis revealed that male reproduction-related pathways were enriched in mutant tumors, whereas WT tumors were more enriched for differentiation and TME-associated pathways such as cancer-associated fibroblasts (CAFs) and myofibroblasts (empowering muscle-like contractions), extracellular matrix (ECM) and immune cells (Supplementary Figure S1D). Together with the GSVA results that multiple stroma-regulatory pathways (e.g. TGFβ, myogenesis, IL-6 and IL-2) were upregulated in WT tumors (Figure 1C), our data implied that m^6^A signaling disturbance caused by genomic alterations may impact PCa progression via, at least partially, reshaping the TME.

Given the determinant role of TILs in both tumorigenesis and anticancer immunity elicited by immunotherapy, we next dissected the tumor immune landscape between the mutant and WT groups by GSVA of a well-established compendium of gene signatures related to specific immune cells (62). Globally, the immune landscape was similar in two groups (Supplementary Figure S1E), with only a limited number of TIL subpopulations being different in abundance (Supplementary Figure S1F). Consistently, calculation of the cancer immunity cycle via a web tool called TIP (see the ‘Materials and Methods’ section) (61) indicated that WT tumors exhibited only a slightly higher activity in step 7 of ‘killing cancer cells’ compared to mutant ones (Supplementary Figure S1G), again indicative of a subtle difference in TIL composition between these two groups.

### Transcriptomic alternations and prognostic values of m^6^A regulators

The CNVs ultimately affect gene expression (6). We next compared the mRNA levels of all m^6^A regulators between normal prostate and pri-PCa tissues in the curated TCGA cohort (11). Among the 24 genes, 11 (including enzymatic writer METTL3) were found upregulated and 3 (including eraser FTO) downregulated in pri-PCa (Figure 2A), in line with the CNV results that many of them were top deleted or amplified (Figure 1B). Moreover, pairwise correlation analysis highlighted a significant, but non-category-specific (i.e. writer, eraser or reader) co-expression pattern among the majority of m^6^A regulators (Supplementary Figure S2A), except that METTL3 negatively correlated with ZC3H13 and erasers (FTO and ALKBH5) due to their downregulation in PCa (Figure 2A). Together, these results indicated, potentially, an overactivation of the m^6^A pathway in PCa. To further explore the clinical relevance of m^6^A regulators, we assessed their prognostic values in patient’s outcome. At individual gene level, univariate Cox regression analysis showed that METTL3, YTHDF1, HNRNPA2B1 and HNRNPC were risk factors, whereas ZC3H13 and FTO were protective factors, of PCa (Supplementary Figure S2B). Survival analysis identified 13 out of 24 as prognostic predictors, with 8 and 5 being classified as unfavorable and favorable genes, respectively (Supplementary Figure S2C). Unfavorable and favorable genes denoted a gene whose higher expression correlated with poor and better patient survival, respectively (6). Unsurprisingly, many of these unfavorable or favorable genes were either up- or downregulated in PCa (Figure 2A). Collectively, these findings implicated a pro-oncogenic role of the aberrant m^6^A pathway in PCa.

### Distinct m^6^A modification patterns classify PCa into three clusters with distinct clinical features

The m^6^A modification pattern (i.e. m^6^A homeostasis) is governed by the expression repertoire of m^6^A regulators (23). Recent studies have reported significant differences between m^6^A modification patterns in different cancer types, where they contributed to tumorigenesis (40,41,65). To further dissect the potential functions played by the m^6^A pathway in PCa, we utilized consensus clustering (based on expression of the 24 regulators) to stratify the curated TCGA cohort (11) into different clusters, with each representing qualitatively a different m^6^A modification status. By trying different *k*-means (Supplementary Figure S2D), three clusters were identified (with a clear trend that many writer and reader genes were upregulated in C3), including 139 patients in cluster 1 (m^6^A_C1), 39 patients in cluster 2 (m^6^A_C2) and 155 patients in cluster 3 (m^6^A_C3) (Figure 2B, left; Supplementary Table S3). Association of clinical features revealed that tumor aggressiveness gradually increased from cluster C1 to cluster C3, in that tumors in C1 and C3 had the lower and higher scores of GS, proportion of advanced tumor stage T3/4, and a better or a worse survival outcome (Figure 2C), respectively. C2 represented a medium cluster with multiple aspects similar to C1, and contained relatively fewer patients; we thus subsequently mainly focused on C1 and C3 for comparisons. As expected, 16 out of 24 m^6^A regulators were found differentially expressed among these three clusters (Figure 2B, right). Notably, and consistent with the prognostic values of m^6^A clusters, two regulators (CBLL1 and FTO) upregulated in C1 had favorable effects, whereas the other eight regulators (METTL3, RBM15B, ELAVL1, FMR1, HNRNPA2B1, HNRNPC, YTHDC1 and YTHDF1) upregulated in C2 or C3 (versus C1) had unfavorable effects, on patient’s survival (Supplementary Figure S2C). Collectively, these results established clinically C3 as the most aggressive, and C1 as the indolent, pri-PCa cluster.

To solidify our findings, we also interrogated the uncurated TCGA pan-cancer PCa cohort (*n* = 494) and observed almost the same results (Supplementary Figure S2E and F). Expression of the 24 regulators well separated patients into three clusters (Supplementary Figure S2E), with C1 and C3 being the least and most aggressive clusters, respectively, in terms of GS, tumor stage and overall survival (Supplementary Figure S2F). Unsurprisingly, 18 out of 24 m^6^A regulators were differentially expressed among these clusters (Supplementary Figure S2E, right). Importantly, 98.7% of samples clustered in C3 in the curated TCGA cohort were also classified in C3 in the uncreated TCGA pan-cancer cohort (Supplementary Figure S2G), validating our strategy of focusing on the curated cohort (11). Moreover, we further extended our analysis to other two PCa datasets (GSE21034 and GSE116918), which had gene expression and clinical information available. For example, in the GSE116918 cohort (*n* = 248), three m^6^A modification patterns were identified (Supplementary Figure S2H) and, again, tumors clustered in C1 and C3 represented the least and worst aggressive ones, respectively (Supplementary Figure S2I). Similar result was also obtained with the Taylor (GSE21034) cohort (Supplementary Figure S2J).

### Molecular pathway characterization of m^6^A clusters

**Figure 3.**
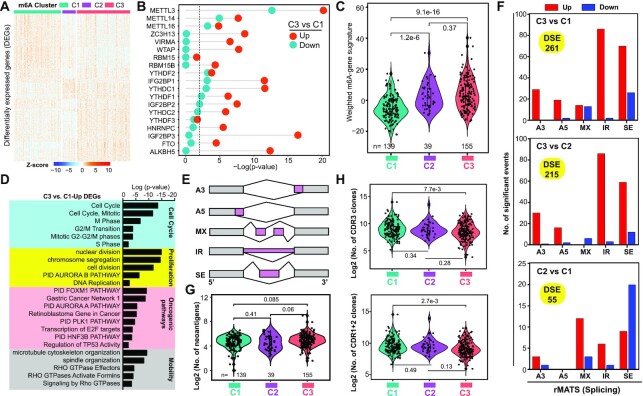
Molecular and immunogenic properties of distinct m^6^A clusters. (**A**) Heatmap of DEGs identified among the three m^6^A clusters in the curated TCGA cohort. (**B**) Overlapping of DEGs identified in C3 versus C1 and potential target genes of distinct m^6^A regulators reported in the m6A2Target database. The *P*-values were calculated using a hypergeometric test. (**C**) Comparison of the scores of a weighted m^6^A gene signature in tumors among the three m^6^A clusters. (**D**) GO analysis of upregulated genes in C3 versus C1 by Metascape. Top enriched GO terms were displayed and grouped into four functional categories (cell cycle, proliferation, oncogenic pathways and mobility). The *P*-values were calculated based on the cumulative hypergeometric distribution. (**E**) Five main types of AS patterns analyzed in the present study. A3, alternative 3′ splice sites; A5, alternative 5′ splice sites; MX, mutually exclusive exons; SE, exon skipping; IR, intron retention. (**F**) Alterations in AS landscape among three m^6^A clusters. Shown are splicing patterns and the number of DSEs decoded by rMATS. (**G**) Number of potential neoantigens generated per tumor in different m^6^A clusters. (**H**) Number of CDR3s (upper) and CDR1/2s (bottom) defined by the TRUST4 algorithm in RNA-seq data. All analyses were based on the curated TCGA cohort. Significance was calculated by the Kruskal–Wallis test (C, G, H).

We next sought to illustrate the transcriptomic differences among the three m^6^A_Clusters (i.e. three different m^6^A modification patterns). By performing the paired DEG analysis, we identified 1082 DEGs in total between any two of these clusters (termed as all DEGs) (Figure 3A; Supplementary Table S4). Among them, 407 genes were commonly dysregulated in at least two comparisons (core DEGs) (Supplementary Figure S3A), and we thus reasoned these 407 genes as m^6^A phenotype-associated genes. Protein–protein interaction analysis indicated that these core DEGs formed an integrated network centered at ACTN2, MYH6, CDK1, TOP2A, ORM1, UTS2B and other genes (Supplementary Figure S3B). GO annotation revealed that this network primarily regulated biological processes linked to TME, metabolism, SC and development, stress response and signal transduction (Supplementary Figure S3C), highlighting a broad impact of m^6^A phenotype-associated genes. Recently, a m6A2Target database was built to report potential targets of 20 m^6^A regulators derived from high-throughput studies (55). Overlapping of DEGs derived from m^6^A_Clusters with genes deposited in the m6A2Target database showed that 47% of all DEGs and 45% of core DEGs were significantly regulated by m^6^A regulators (hypergeometric test, Supplementary Figure S3D). Focusing on the two extreme clusters, pair comparison of C1 and C3 identified 460 DEGs (Supplementary Table S4). Interestingly, overlapping of them with the potential targets of individual m^6^A regulators indicated that 240 upregulated genes in C3 were more likely regulated by m^6^A regulators compared with the 220 downregulated genes (Figure 3B; Supplementary Table S5). This was consistent with the fact that the m^6^A pathway was more activated in C3, reflected by the higher signature score of weighted expression of 24 m^6^A regulators (Figure 3C). Moreover, functional annotation showed that the upregulated genes in C3 were mainly enriched in tumor-promoting pathways, such as cell cycle progression and proliferation, multiple known oncogenic pathways and cell migration (Figure 3D), consistent with a more aggressive phenotype of C3 over C1. Interestingly, the relatively indolent C1 tumors were primarily driven by pathways associated with TME, metabolism, differentiation and cell adhesion (Supplementary Figure S3E). Particularly, TME was the most enriched category in low-m^6^A-activity C1, again suggesting a pro-oncogenic role for m^6^A signaling via negatively shaping TME (also see later). Notably, C2 was nonsignificantly different from C1 in both survival and tumor stage analysis (Figure 2C); we thus observed subtle difference between up- and downregulated DEGs being identified as potential targets of m^6^A regulators (Supplementary Figure S3F and Supplementary Table S5). Currently, there is little study examining the global m^6^A modification landscape in clinical PCa specimens; hence, m6A2Target contained none PCa-relevant data. To further strengthen our findings with m6A2Target analysis, we performed, preliminarily, m^6^A-seq in three PCa tissues and identified a total of 14 354 genes bearing m^6^A peaks. Overlapping of DEGs between C3 and C1 with this clinically relevant m^6^A targets showed a 34.13% overlap (Supplementary Figure S3G), a percentage close to an estimated ∼40% of human genes that can be m^6^A modified (55).

### Splicing and immunogenic properties of distinct m^6^A modification patterns

Splicing dysregulation is a hallmark of cancer (6,66). We have recently demonstrated that the severity of splicing abnormalities correlates with disease progression, and established intron retention (IR) as a hallmark of PCa stemness and aggressiveness (6). We next performed AS analysis between any two of the three m^6^A clusters (Figure 3E), and defined a total of 261, 215 and 55 DSEs in comparisons of C3 versus C1, C3 versus C2 and C2 versus C1, respectively (Figure 3F). Particularly, more AS events (especially the IR and skipping exon) were found in C3 relative to C1 or C2 (Figure 3F; Supplementary Figure S3H), potentially leading to generation of neoantigens. Using a recently published cancer antigenome across TCGA solid cancers including PCa (62), we found a trend, although not significant (*P* = 0.06), that the number of neoantigens increased along with tumor progression from C1 to C3 (Figure 3G). Neoantigens are attractive candidates for developing cancer vaccines, but their recognition by the immune system depends on efficient presentation. Due to a lack of global characterization of neoepitopes in TCGA-PRAD samples, we took another computational approach to infer neoantigen load in m^6^A_Clusters by reconstituting both the B- and T-cell receptor repertoires via a newly developed TRUST4 algorithm (67). To our surprise, the number of complementary-determining region 3 (CDR3) clones decreased from C1 to C3, so as the CDR1 and CDR2 on the V sequence (Figure 3H). This discrepancy between a higher number of potential neoantigens and a lower number of immune receptor repertoires highlighted a defect in the antigen presentation process in C3 tumors. In support, GSVA of four extracted signatures of antigen presentation indicated that they were all gradually decreased from C1 to C3 (Supplementary Figure S3I), in line with the TME analysis presented later.

### Different m^6^A modification patterns characterized by cancer hallmarks and TME

**Figure 4.**
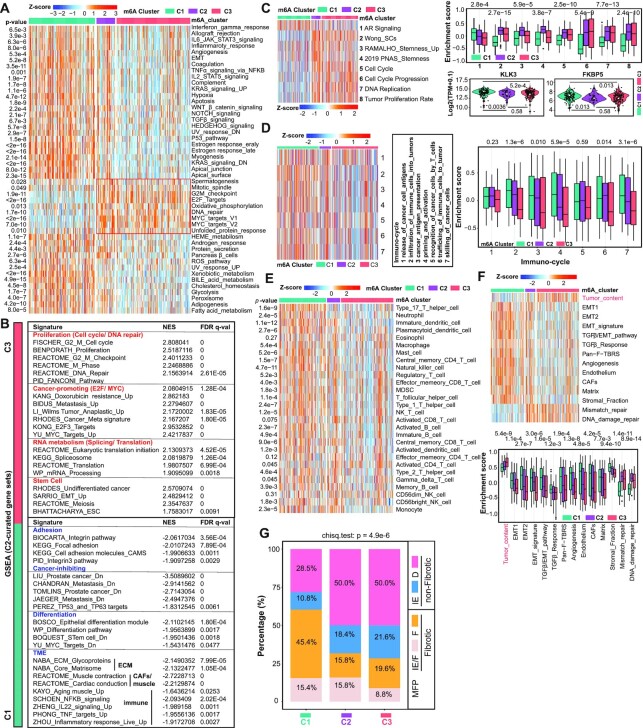
Different m^6^A modification patterns characterized by cancer hallmarks and TME. (**A**) Heatmap showing enrichment of the representative cancer hallmarks from MSigDB by GSVA in three m^6^A clusters in the curated TCGA cohort, with *P*-values labeled on the left. (**B**) Comparative GSEA of transcriptomes in C3 versus C1 showing C3 as a more proliferative and stem-like cluster and C1 as a more differentiated, less migrative and stroma-enriched cluster. The FDR for GSEA is the estimated probability that a gene set with a given NES represents a false-positive finding and an FDR < 0.25 is considered to be statistically significant. (**C**) Pathway analysis of different m^6^A modification patterns. Heatmap presentation (left) and boxplot quantification (right, upper) of the enrichment of indicated signatures among three m^6^A clusters. The expression dynamics of two known AR target genes (KLK3 and FKBP5) are also shown (right, bottom). (**D**) Cancer immunity cycle analysis by GSVA showing C1 and C3 as the clusters with the highest and the lowest anticancer immunity, respectively. (**E**) Heatmap showing enrichment of the 28 TIL subpopulations calculated by GSVA in three m^6^A clusters, with *P*-values labeled on the left. Red and blue in scale bar denote relatively high and low abundances of subpopulations, respectively. (**F**) Heatmap presentation (upper) and boxplot quantification (bottom) of the enrichment of indicated signatures among three m^6^A clusters. Within the plots, the center lines represent median values, box edges are 75th and 25th percentiles, and dots denote the outliers. (**G**) Distribution of four previously reported TME subtypes in three m^6^A lusters, with *P*-value (chi-square test) labeled on top. IE/F: immune-enriched, fibrotic; IE: immune-enriched, nonfibrotic; F: fibrotic; D: immune-depleted. IE/F and F are fibrotic, while IE and D are nonfibrotic. All analyses were based on the curated TCGA cohort (*n* = 333), and significance was all calculated by the Kruskal–Wallis test (A, C–F).

Next, GSVA enrichment analysis was performed against the hallmark gene set in MSigDB to comprehensively dissect the biological properties associated with the three m^6^A_Clusters. A number of protumorigenic signatures (e.g. MYC targets, DNA repair, E2F targets and G2M checkpoint) were significantly enriched in C2 and C3 (Figure 4A); however, paradoxically, some antitumor pathways (e.g. p53 pathway, apoptosis and reactive oxygen species pathway) were overrepresented in C1, consistent with the overmentioned results that C1 and C3 were the least and most aggressive clusters, respectively. Interestingly, multiple immune-related (e.g. IFNγ response, IL-6, IL-2 and TNF-α signaling) and differentiation/metabolism-related (e.g. estrogen/androgen response, protein secretion and myogenesis) signatures were exclusively enriched in C1 (Figure 4A). To further confirm these findings, we extended our analysis to GSEA of 6290 curated gene sets in MSigDB against comparative transcriptomes of C3 versus C1, and found that pathways related to proliferation, cancer promotion, SCs and RNA metabolism were significantly enriched in C3, whereas pathways related to adhesion, cancer inhibition, differentiation and TME were overrepresented in C1 (Figure 4B). Notably, these results were generally in line with GO analysis of the limited number of DEGs (Figure 3D; Supplementary Figure S3E). AR is obligatory for pri-PCa growth and continues to be expressed and functionally important in CRPC (68). ADT promotes stemness (69) and CRPCs are generally stem-like regardless of AR expression status (3). Also, canonical AR transcriptional activity decreases along with tumor progression (6,9). We next performed a focused GSVA on relevant pathways. As expected, compared to C1, clusters C2 and C3 were more stem-like [evidenced by enrichment of three (2-4) stemness signatures] and proliferative [evidenced by enrichment of four (5-8) proliferation signatures] (Figure 4C). Of note, examination of an AR signature (a panel of 30 genes that were previously reported as defining the pathway) (57) and two well-known AR target genes (KLK3 and FKBP5) clearly demonstrated that AR activity was gradually decreased from C1 to C3 (Figure 4C). Collectively, these data defined C1 as AR^high^ and relatively indolent cluster and C2/C3 as AR^low^ stem-like and highly proliferative clusters.

Due to our frequent observation that the deregulated m^6^A modification patterns impacted TME (especially the immune category) the most (Figure 4B; Supplementary Figures S1D and S3E), we next focused on the immune landscape. Globally, multiple immune-activating processes (e.g. inflammatory response, IFNγ response, allograft rejection and TNF-α signaling) were exclusively enriched in C1 (Figure 4A and B). Consistently, cancer immunity cycle analysis revealed that C1 displayed prominently higher activity over C2 and C3 in five out of seven immune steps, including infiltration of immune cells into PCa, cancer antigen presentation, trafficking of immune cells and killing of cancer cells (Figure 4D). In support, ESTIMATE quantification of the overall TILs showed that C1 had the highest ImmuneScore (Supplementary Figure S4A). To gain details in cellular composition of TME, we quantified the abundance of 28 TIL subpopulations based on previously established cell type-specific signatures (62). As showed in Figure 4E, 24 out of 28 immune subsets were significantly different in abundance among the three m^6^A_Clusters. In particular, C1 and C3 had the highest and lowest abundances in 16 and 23 immune subsets, respectively (Figure 4E). For example, C1 and C2 exhibited higher scores for antitumor subsets compared to C3, including activated B and dendritic cells, central memory CD4 T cells, effector memory CD8 T cells, natural killer (NK) cells and NK T cells (Figure 4E). Notably, some immunosuppressive subsets also appeared in high abundance in C1 and C2 (e.g. immature dendritic cells, monocytes, neutrophils, regulatory T cells and type 2 T helper cells), which could be explained by a global increase in TILs in these clusters.

Besides cancer and immune cells, TME also contains surrounding blood vessels, fibroblasts and the ECM (70). We noticed that tumors in C3 cluster had the highest tumor purity followed by C2 and C1 clusters (Figure 4F), implying an opposite proportion of stroma in these clusters. In support, the StromaScore (63) gradually decreased from C1 to C3 (Supplementary Figure S4B), and the stroma activity was high in C1 (followed by C2 and C3), evidenced by elevated enrichment of stroma-related pathways such as epithelial–mesenchymal transition, pan-fibroblast TGFβ response signature, angiogenesis, CAFs and matrix (Figure 4F). We thus defined C1 as a high-fibrotic cluster, whereas the other two as low-fibrotic clusters. Recently, a pan-cancer analysis has categorized >10 000 tumors in TCGA into four TME subtypes (based on a set of 29 knowledge-based functional gene expression signatures): immune-enriched and fibrotic (IE/F), immune-enriched but nonfibrotic (IE), fibrotic (F) and immune-depleted (D) (71). Accordingly, distribution of our m^6^A_Clusters indicated that the fibrotic (IE/F and F) proportion of tumors classified in C1 (60.8%) was approximately twice as much as that in C2 (31.6%) or C3 (28.4%) (Figure 4G), supporting our GSVA results (Figure 4F). It was worth noting that the DNA repair activity was significantly high in C2 and C3 compared with C1 (Figure 4F), indicative of proliferation and consistent with cancer hallmark analysis (Figure 4A). Importantly, and expectedly, a detailed interrogation of the uncurated TCGA pan-cancer cohort generated similar results. For instance, 19 out of 28 immune subsets were different in their abundance among m^6^A_Clusters, with a clear decreasing trend from C1 to C3 (Supplementary Figure S4C). Consistently, five out of seven anticancer immune steps were low in activity in C3 relative to C1 (Supplementary Figure S4D). Pathway analysis indicated a more stem-like and proliferative phenotype for C2 and C3 (Supplementary Figure S4E), whereas C1 was more fibrotic (Supplementary Figure S4F).

### Construction of m^6^Avalue based on m^6^A phenotype-associated genes

**Figure 5.**
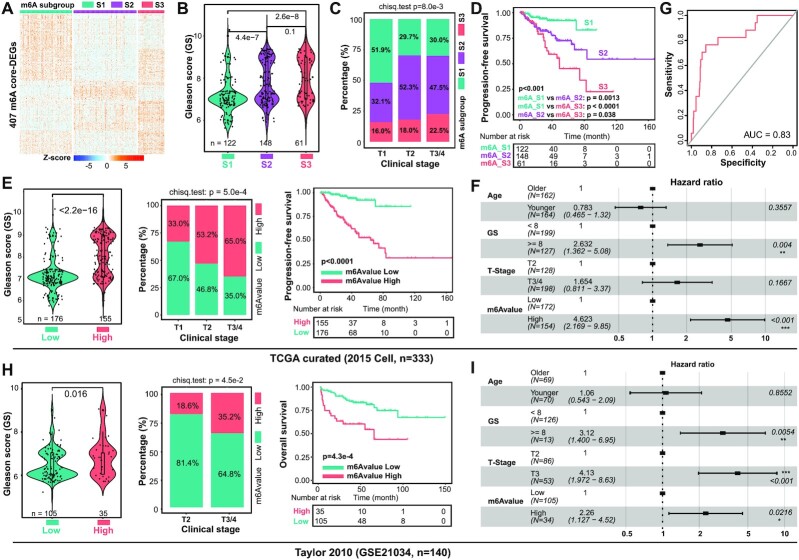
m^6^Avalue clearly stratifies PCa patients with distinct clinical outcomes. (**A**) The expression repertoire of 407 core DEGs classifies pri-PCa into three subgroups (m^6^A_S1/2/3). Comparison of GS (**B**), tumor stage (**C**) and patient’s survival outcome (**D**) showing S1 and S3 being the least and most aggressive PCa subgroups, respectively. The proportion of patients clustered into m^6^A_S2/3 in advanced T2 (52.3% + 18.0% = 70.3%) and T3/4 (47.5% + 22.5% = 70%) stages is much higher than that in T1 (32.1% + 16.0% = 48.1%) stage (C). Significance was calculated by the Kruskal–Wallis test (B), chi-square test (C) and long-rank test (D). (**E**) Comparison of GS (left), tumor stage (middle) and patient’s survival outcome (right) showing the m^6^Avalue^high^ group being more aggressive. Significance was calculated by the Wilcoxon test (left), chi-square test (middle) and long-rank test (right). (**F**) Multivariate Cox regression analysis showing m^6^Avalue as an independent prognostic factor among other indicated clinical parameters. The length of the horizontal line represents the 95% confidence interval for each group. The vertical dotted line denotes the HR of all patients. (**G**) The predictive value of m^6^Avalue in the curated TCGA cohort measured by ROC curves. ROC, receiver operating characteristic; AUC, area under the curve. (**H** and **I**) Validation in an independent Taylor dataset. Comparison of GS (H, left, Wilcoxon test), tumor stage (H, middle, chi-square test) and patients’ survival outcome (H, right, long-rank test) showing the m^6^Avalue^high^ group being more aggressive. Notably, 10 patients without full information of GS, clinical stage and survival time were omitted. Multivariate Cox regression analysis showing m^6^Avalue as an independent prognostic factor among other indicated clinical parameters (I).

Considering the intratumoral heterogeneity in PCa and the conservation of m^6^A-mediated biology in a given tumor type, and to further dissect the m^6^A-associated phenotypes, we performed consensus clustering analysis on 407 core DEGs (Supplementary Figure S5A) to optimally classify tumors into three distinct subgroups (Figure 5A), namely m^6^A_S1 (124 patients), m^6^A_S2 (148 patients) and m^6^A_S3 (61 patients), respectively. In line with the m^6^A_Clusters (Figure 2C), the aggressiveness of m^6^A_Subgroups increased from S1 to S3, evidenced by gradually elevated GS (Figure 5B), advanced tumor stage (Figure 5C) and worse survival outcome (Figure 5D). Expectedly, most of the m^6^A regulators were dysregulated among these subgroups (Supplementary Figure S5B), and the patient distribution in m^6^A_Subgroups was in high concordance with that in m^6^A_Clusters (especially the S1 and S3 to C1 and C3, correspondingly) (Supplementary Figure S5C). Molecular interrogation of m^6^A_Subgroups revealed, generally, similar results with what was observed in m^6^A_Clusters (Figure 4), in terms of cancer hallmarks (Supplementary Figure S5D), stemness/proliferation/stroma-related signatures (Supplementary Figure S5E), cancer immune cycle analysis (Supplementary Figure S5F) and the 28 immune subpopulations (Supplementary Figure S5G). Briefly, compared to S2 and S3, the S1 subgroup was relatively indolent with obvious enrichment of stroma- and immune-related pathways, 22 of 28 TIL subpopulations, higher AR activity and anticancer immunity.

Population-based classifiers cannot directly apply to individual patients for predicting a pattern of m^6^A methylation landscape. To facilitate a potential clinical use of our PCa classifications, we constructed m^6^Avalue, a scoring system collectively quantifying the m^6^A modification patterns based on a weighted 87-gene signature shrank down from the 407 m^6^A-associated genes. These 87 genes were selected due to their association with clinical patient survival (see the ‘Materials and Methods’ section). Calculation of m^6^Avalue in m^6^A_Clusters and m^6^A_Subgroups showed that it increased along with tumor aggressiveness from C1 to C3 or S1 to S3 (Supplementary Figure S5H), validating our algorithm. Unsurprisingly, m^6^Avalue separated pri-PCa into two groups, with the m^6^Avalue^high^ (relative to m^6^Avalue^low^) group being more aggressive (Figure 5E). Multivariate Cox regression analysis confirmed that m^6^Avalue could serve as an independent prognostic biomarker for patient outcomes, among other clinical parameters (Figure 5F). In support, ROC curve further demonstrated the predictive accuracy of m^6^Avalue (Figure 5G). Besides the curated TCGA cohort, we also validated our m^6^Avalue model in other two independent datasets, GSE21034 (Figure 5H and I) and GSE116918 (Supplementary Figure S5I and J). In both datasets, m^6^Avalue could function as an independent prognostic factor, and the m^6^Avalue^high^ (relative to m^6^Avalue^low^) group was correlated with adverse tumor grade and worse prognosis, suggesting the robustness of our m^6^Avalue model. Importantly, albeit our findings were made on pri-PCa due to the intrinsic data properties, paired comparison indicated that the m^6^Avalues in CRPC samples (12) were significantly higher than those in pri-PCa samples (Supplementary Figure S5K), further strengthening the idea that m^6^Avalue^high^ tumors are more aggressive (and perhaps treatment resistant).

### m^6^Avalue stratifies PCa with distinct molecular and phenotypic characteristics

**Figure 6.**
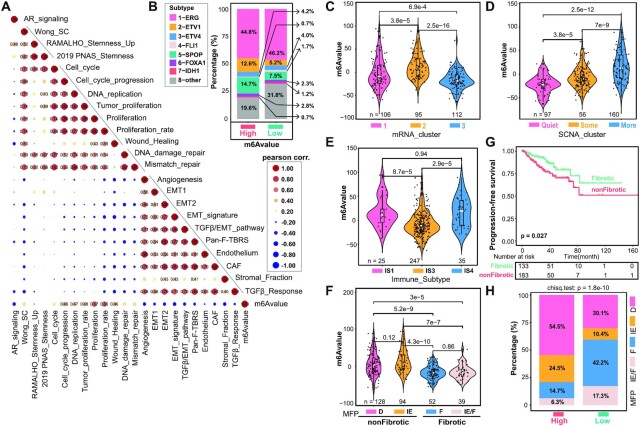
m^6^Avalue separates TME into fibrotic and nonfibrotic phenotypes. (**A**) Pearson correlation of m^6^Avalue with indicated signatures. Positive and negative correlations are colored in red and blue, respectively. (**B**) Comparison of the eight prevalent PCa genomic subtypes reported by TCGA (11) showing similar patterns between the m^6^Avalue^high^ and m^6^Avalue^low^ groups. Violin plot showing difference in m^6^Avalue of tumors classified in TCGA mRNA_cluster (**C**) and SCNA_cluster (**D**) (11), recently reported Immune_Subtype (**E**) (72) and TME subtype (**F**) (71). Significance was calculated by the Kruskal–Wallis test. (**G**) The Kaplan–Meier plot showing a worse survival outcome associated with the nonfibrotic phenotype. The *P*-value was calculated using the long-rank test. (**H**) Distribution of four previously reported TME subtypes in the m^6^Avalue^high^ and m^6^Avalue^low^ groups with *P*-value (chi-square test) labeled on top. Prostate tumors with higher m^6^Avalues tend to exhibit nonfibrotic phenotype (79.0% versus 40.5%). IE/F: immune-enriched, fibrotic; IE: immune-enriched, nonfibrotic; F: fibrotic; D: immune-depleted. IE/F and F are fibrotic, while IE and D are nonfibrotic. All analyses were performed based on the curated TCGA cohort (*n* = 333).

To better illustrate the characteristics of m^6^Avalue, we first performed a global correlation analysis between m^6^Avalue and a selected set of key signatures/pathways. As shown in Figure 6A, m^6^Avalue was positively correlated with the activity of stemness, proliferation and DNA repair, while negatively correlated with AR and stroma-centered signatures. Notably, m^6^Avalue was also negatively correlated with the anticancer immunity and abundance of the majority of 28 immune subpopulations in pri-PCa (Supplementary Figure S6A), establishing again m^6^Avalue as a predictor of low immunity in TME. Previously, the TCGA landmark paper (11) has categorized pri-PCa into three clusters based on tumor transcriptomes (mRNA clusters 1–3), and reported that, genomically, 74% of pri-PCa belonged to either one of seven subtypes defined by gene fusions (ERG, ETV1/4 and FLI1) or mutations (SPOP, FOXA1 and IDH1). In our analysis, we found that the distribution of these genomic subtypes was similar in the m^6^Avalue^high^ and m^6^Avalue^low^ groups (Figure 6B), except that the m^6^Avalue^high^ group contained a bit higher fraction of tumors bearing ETV1 fusion and SPOP mutation. Furthermore, the TCGA mRNA cluster 3 had the lowest m^6^Avalue (Figure 6C), and consistently had a better prognosis (Supplementary Figure S6B). The somatic CNV (SCNV) is associated with PCa recurrence and metastasis, and pri-PCa have been clustered previously into three clusters (Quiet, Some and More) based on the SCNV burden (11). Interestingly, we found that m^6^Avalue was positively correlated with SCNV burden (Figure 6D), in line with enhanced genome instability and, simultaneously, DNA repair activity in advanced PCa.

In a recent pan-cancer study (72), all human tumors regardless of origin can be categorized into six immune subtypes (IS1–IS6), and PCa mainly fall into IS1, IS3 and IS4. Notably, the IS1 was characterized by elevated expression of angiogenic genes and high proliferation rate; IS3 by low proliferation and SCNV burden but with high Th17 and Th1 activity; and IS4 by a prominent macrophage signature and a repressed Th1 activity (72). Particularly, IS3 correlated with longer survival time in multiple cancer types, including PCa (72). When we compared the m^6^Avalue in these immune subtypes, we found that IS3 and IS4 had the lowest and highest m^6^Avalues (Figure 6E) and, consistently, a better and a worse prognosis, respectively (Supplementary Figure S6C). Interestingly, the difference in m^6^Avalue among pri-PCa specimens was fibrotic phenotype specific (similar m^6^Avalue for IE/F and F) rather than immune-enriched phenotype specific (unsimilar m^6^Avalue for IE/F and IE), with nonfibrotic phenotypes possessing higher m^6^Avalues (Figure 6F). Accordingly, when we regrouped the pri-PCa into fibrotic (F and IE/F) and nonfibrotic (IE and D) subgroups, we found that the nonfibrotic subgroup was more aggressive, evidenced by the higher GS and tumor stage (Supplementary Figure S6D) and a worse survival outcome (Figure 6G). In support, the m^6^Avalue^high^ group had a high proportion of tumors with nonfibrotic phenotype (79% versus 40.5%; Figure 6H), indicative of fibrotic phenotype as a marker for indolent PCa.

### The m^6^Avalue correlates with therapeutic effects of small-molecule inhibitors

**Figure 7.**
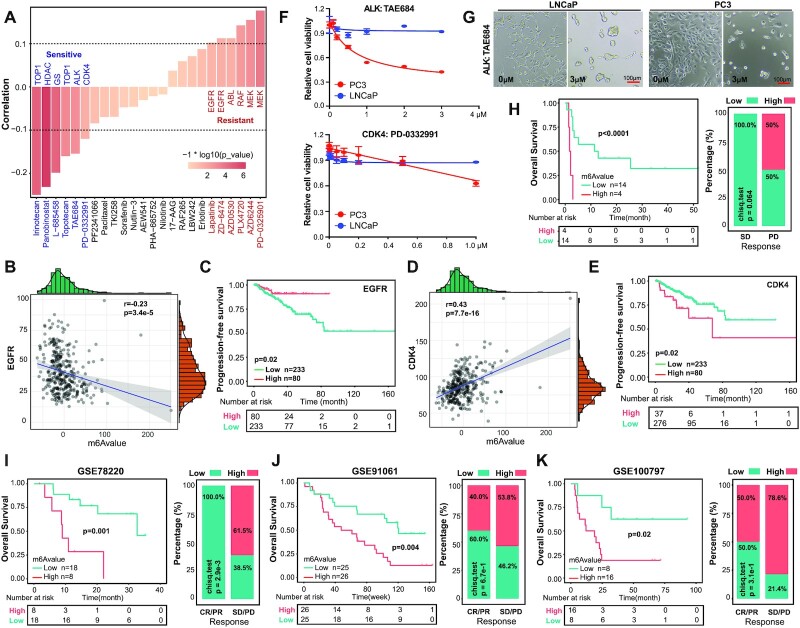
The m^6^Avalue predicts responses to both small-molecule inhibitors and immunotherapies. (**A**) Spearman correlation of m^6^Avalue with IC_50_ of different compounds reported in the CCLE database. Pairwise results were filtered by correlation >0.1 or <−0.1 with *P* < 0.05. Compounds showing positive and negative correlations with m^6^Avalue were considered as resistant (red) and sensitive (blue) drugs. Pearson correlation of expression of EGFR (**B**) or CDK4 (**D**) with m^6^Avalue in the curated TCGA cohort. The upper and right margins denote the distribution of gene expression and m^6^Avalue, respectively. Kaplan–Meier analysis showing high levels of EGFR (**C**) and CDK4 (**E**) being correlated with a better and a worse patient survival, respectively. (**F**) Cell viability (MTT) assay in indicated cells treated with TAE684 (top) or PD-0332991 (bottom) for 4 days. Data represent mean ± SD from a representative experiment with four technical repeats and the experiment was replicated two times with similar results. (**G**) Morphological changes of indicated cells treated with TAE684 for 4 days. Scale bar, 100 μm. The low m^6^Avalue is associated with a better response to immunotherapies in an anti-CTLA-4 CRPC cohort (**H**) and three independent melanoma cohorts [GSE78220 (anti-PD-1, **I**), GSE91061 (anti-PD-L1, **J**) and GSE100797 (adoptive T-cell therapy, **K**)]. Shown are survival analysis (left) and the fraction of patients with clinical responses to treatment (right) in low or high m^6^Avalue groups for each cohort. CR, complete response; PR, partial response; SD, stable disease; PD, progressive disease. Significance for survival analysis and patient fraction distribution were calculated by the long-rank test and chi-square test, respectively.

Inspired by the crosstalk between m^6^Avalue and many vital cancer-related pathways, together with an aim to extend the potential usage of m^6^Avalue in therapeutic settings, we next explored whether the intrinsic m^6^Avalue of cancer cells predicts drug response. Utilizing the pan-cancer CCLE database (73), we calculated the m^6^Avalue for each cell line and identified 12 significant correlations (Figure 7A). Specifically, the IC_50_ values of six drugs targeting EGFR, ABL, RAF and MEK were positively correlated with m^6^Avalue (indicating drug resistance), whereas the IC_50_ values of another six drugs targeting TOP1, HDAC, GS, ALK and CDK4 were negatively correlated with m^6^Avalue (indicating drug sensitivity) (Figure 7A), pointing potential therapeutic strategies that aggressive PCa might be more sensitive to these drugs. Although the upregulation of EGFR has been observed in, and causally associated with, many cancers (74,75), our data showed that m^6^Avalue negatively correlated with EGFR expression in pri-PCa (Figure 7B) and, consistently, patient’s tumor with higher expression of EGFR had lower GS and tumor stage (Supplementary Figure S7A) and ultimately a better prognosis (Figure 7C). These results urged a cancer type-specific effect of EGFR expression and thus EGFR inhibitors on tumorigenesis. CDK4 plays an essential role in cell cycle progression and inhibitors of CDK4/6 have been broadly used as an antitumor strategy (76). In pri-PCa, the m^6^Avalue was significantly and positively correlated with CDK4 expression (Figure 7D). As expected, CDK4 played an oncogenic role in PCa as its higher expression predicted higher GS and tumor stage (Supplementary Figure S7B), and a worse outcome (Figure 7E). To provide proof-of-principle evidence, we experimentally tested the cell growth inhibitory effect of ALK (TAE684) and CDK4 (PD-0332991) inhibitors in well-known indolent LNCaP and aggressive PC3 lines (6), finding that PC3 (versus LNCaP) cells were more sensitive to these targeted therapies (Figure 7F). Interestingly, we observed that TAE684 induced cell aggregation in LNCaP, while cell death in PC3, cells (Figure 7G).

### The m^6^Avalue predicts immunotherapeutic efficacy

Having established a direct link between the global m^6^A modification pattern and immune landscape in PCa in our multilayer analyses, we next sought to define whether m^6^Avalue could predict patients’ responses to immunotherapy. In PCa, datasets with available information of both clinical responses to immunotherapies and survival/gene expression data were scanty. Recently, a clinical trial with anti-CTLA-4 (i.e. ipilimumab) in 30 patients with metastatic CRPC was conducted, with stable disease and progressive disease being defined as beneficial and nonbeneficial responses, respectively (44). When we stratified the small cohort based on m^6^Avalue, we found that the m^6^Avalue^low^ group survived better and had a higher proportion of patients with stable disease (Figure 7H). To further validate our results, we utilized another three melanoma cohorts treated with different ICBs. In two anti-PD-1 datasets (GSE78220 and GSE91061), m^6^Avalue well separated the cohort into two groups (Supplementary Figure S7C) and patients with lower m^6^Avalues responded better to immunotherapy and thus survived longer (Figure 7I and J). Comparison of the well-known immunotherapeutic targets (i.e. PD-1, PD-L1 and CTLA-4) showed similar expression patterns, with PD-L1 tending to upregulate in the m^6^Avalue^low^ versus m^6^Avalue^high^ group (Supplementary Figure S7D). Examination of another adoptive T-cell therapy cohort (GSE100797) generated similar results (Figure 7K; Supplementary Figure S7E), except that both PD-L1 and CTLA-4 were significantly overexpressed in the m^6^Avalue^low^ group (Supplementary Figure S7F). Collectively, our data strongly established m^6^Avalue as a reliable biomarker predicting immunotherapy response in multiple cancer types.

## DISCUSSION

As the most abundant chemical modification present in multiple RNA species (especially mRNA), m^6^A plays key roles in almost every aspect of RNA metabolism, as well as in a variety of physiological and pathological processes (22). Although there is limited evidence (based on individual gene studies) implicating m^6^A regulators in PCa biology, study of the m^6^A pathway in PCa generally lags when compared to other cancer types (e.g. leukemia and breast) (77). Currently, a comprehensive analysis that integrates the full set of recognized m^6^A regulators that better reflects the m^6^A methylation patterns in PCa is lacking. In this study, by comprehensively annotating the genomic and transcriptomic alterations of 24 m^6^A regulators and the repertoire of m^6^A phenotype-associated genes in pri-PCa, we have made several significant (and PCa-specific) findings (also see discussion in the Supplementary Data). First, m^6^A regulators constitute a frequently mutated pathway (albeit a very low alteration frequency at individual gene level) at the population level, indicating an involvement of m^6^A in PCa. Notably, the mutational landscape of m^6^A regulators fails to stratify PCa dramatically, suggesting mutation in m^6^A regulators as a nonsignificant mechanism (versus gene expression regulation) driving tumorigenesis. Second, we unveil that many m^6^A regulators are not only differentially expressed in PCa (versus normal tissues) but also prognostic, highlighting a utility of m^6^A regulators as prognostic biomarkers. Strikingly, the expression repertoire of 24 regulators elegantly classifies TCGA-PRAD and other three independent cohorts into three clusters with distinct molecular and clinical features. In particular, m^6^A_C1 and m^6^A_C3 are, comparatively, the indolent and progressive clusters, respectively. We also demonstrate the superior design of our classification by cross-comparison with previously reported classifications [such as TCGA (11)]. Third, molecular characterization of m^6^A_Clusters reveals that m^6^A methylation modification patterns predominantly impact TME, especially the immune landscape. Both the anticancer immunity and abundance of 28 TIL subsets are significantly higher in C1 than in C3, indicating an inflamed TME for C1 tumors. Interestingly, unlike studies in colon cancer showing that previously well-recognized three immune profiles (i.e. immune-inflamed, immune-excluded and immune-desert) distinguish TME (41), our results indicate that fibrotic and nonfibrotic phenotypes, instead, better depict PCa TME. In support, colon tumors with activation of stroma-related signatures were classified as immune-excluded and linked to poor prognosis. However, we find that prostate tumors in m^6^A_C1 with significant enrichment of these signatures conversely have a better outcome. Fourth, and for the first time, our splicing and immunogenicity analyses of different m^6^A methylation modification patterns highlight a defect in the antigen presentation process in aggressive PCa, which accounts for the discrepancy between a higher number of potential neoantigens and a lower level of immune recognition in C3 versus C1 clusters. These data imply a therapeutic use of splicing inhibitors and ICB for treating aggressive C3 tumors. This concept has been recently validated in murine cancer models (78). Fifth, a negative correlation of AR activity with m^6^A activity is noticed, and together with a recent report that AR is not a direct target of METTL3 (79), we propose AR and m^6^A axis as independent contributors in driving PCa progression. Sixth, m^6^Avalue, a weighted score established from 87 key m^6^A-associated genes, well separates indolent pri-PCa from aggressive ones, with the m^6^Avalue^high^ group being more stem-like, proliferative and nonfibrotic. Seventh, the distorted m^6^A signaling likely contributes to anti-androgen treatment failure and PCa progression via TME reshaping, as CRPCs exhibit globally higher m^6^Avalues than pri-PCa and, relatively, m^6^Avalue^low^ CPRC patients respond better to ICB. These results establish m^6^Avalue as a valuable guide for decision-making on usage of immunotherapy (especially considering that there are currently no established biomarkers for immunotherapeutic efficacy). Finally, a pan-cancer analysis indicates that m^6^Avalue can be used to predict sensitivity of small-molecule-based targeted therapy. Accordingly, we hypothesize that aggressive PCa with high m^6^Avalue might be more vulnerable to inhibitors of CDK4, ALK, TOP1 and others, directing novel therapeutic strategies that warrant further exploration. Future in-depth characterizations of individual m^6^A regulators, together with the kinetics of cellular m^6^A levels, in PCa etiology and progression, could enhance our understanding of disease pathogenesis and aid innovative drug development.

## DATA AVAILABILITY

As detailed in the ‘Materials and Methods’ section, all data used for main bioinformatic analysis are publicly available.

## Supplementary Material

zcac010_Supplemental_Files
