# Supplementary material for: A m6Avalue predictive of prostate cancer stemness, tumor immune landscape and immunotherapy response
Source: NAR Cancer. 2022 Mar 25;4(1):zcac010. doi: 10.1093/narcan/zcac010 (PMC8953419; doi:10.1093/narcan/zcac010)
Supplement: zcac010_Supplemental_Files [file zcac010_supplemental_files.zip › Supplementary Data.docx]

**A m^6^Avalue predictive of prostate cancer stemness, tumor immune landscape and immunotherapy response**

Cheng Zou^1,2^, Qinju He^1^, Yuqing Feng^1^, Mengjie Chen^1^, Dingxiao Zhang^1*^

^1^School of BioMedical Sciences, Hunan University, Changsha 410082, China

^2^College of Animal Science and Veterinary Medicine, Huazhong Agricultural University, Wuhan 430070,

China

*Correspondence: [zdx1980@hnu.edu.cn](mailto:zdx1980@hnu.edu.cn)

**Supplementary Data**

1. Supplementary Discussion

2. Supplementary Figures

3. Supplementary Tables

**1. Supplementary Discussion**

The m6A modification plays important roles in tumor development and progression through various mechanisms ([1](#_ENREF_1),[2](#_ENREF_2)). Particularly, individual m6A regulators have been reported to exhibit contradictory, even opposing, roles (e.g., oncogene or tumor suppressor) in distinct cancer types, complicating the development of potential m6A pathway targeted therapies ([3-5](#_ENREF_3)). To reveal the potential role of m6A in PCa, here we comprehensively analyze the m6A pathway from multiple angles. By utilizing similar bioinformatic methodology, several recent publications, such as pan-cancer ([6](#_ENREF_6),[7](#_ENREF_7)) and cancer-type specific (e.g., pancreatic ([8](#_ENREF_8)), gastric ([9](#_ENREF_9)) and colon cancer ([10](#_ENREF_10))) studies, have suggested that m6A modification patterns can molecularly classify patients into different clusters with varying prognostic features. In pan-cancer analyses, the general portrait of genomic and transcriptional alterations in m6A regulators cross 30 caner types, as well as their relationship with patient outcomes, has been summarized ([6](#_ENREF_6),[7](#_ENREF_7)). However, given the intrinsic difference in each cancer type, a tailored PCa-focused analysis is still worth exploring and important for new drug development. In cancer-type specific analyses (this study and others ([8-10](#_ENREF_8))), although similar analytic approaches are used, we do find many findings that are PCa-specific and distinct to other cancer types. For example, unlike the abovementioned studies ([8-10](#_ENREF_8)) that reveal immune profile (i.e., immune-inflamed/immune-excluded/immune-desert) as the determinant for defining TME in different m6A clusters in other cancer types, we find that, interestingly, fibrotic and nonfibrotic phenotypes are better biomarkers to profile PCa TME. Importantly, in contrast to the oncogenic role of fibrotic phenotype in several other cancer types (i.e., esophagogastric, liver cancer, and lung cancer) ([11](#_ENREF_11)), our data indicated that it correlates with better outcomes in PCa (Fig. 6G), highlighting a tumor-type specificity. Furthermore, linking TME features to patient outcomes, studies in pancreatic ([8](#_ENREF_8)), gastric ([9](#_ENREF_9)) and colon cancer ([10](#_ENREF_10)) were mainly focused on the TILs density. In this study, besides this point, we have expanded our analysis to look at several other facets, such as the cancer-immunity cycles and many clinically PCa-relevant pathways (i.e., AR activity, MYC and cancer stemness). More cancer-type specific information will, in general, instruct us better understand the potential mechanisms underlying the impact of m6A pathway on tumor progression. Additionally, we have for the first time correlated m6A modification patterns with splicing dysregulation in PCa and applied a newly developed algorithm, TRUST4 ([12](#_ENREF_12)), to uncover a potential higher load of neoantigens in aggressive C3 vs. relatively indolent C1 (Fig. 3G). Together with cancer-immune cycle analysis, we propose a gap between more splicing alterations (potentially leading to more neoantigens) and lower immunogenicity of aggressive (vs. indolent) PCa, which may be caused by defects in antigen-presentation process in aggressive tumors. Resolve of this question may yield novel immunotherapy strategies for treating aggressive PCa subtypes. Finally, and importantly, we not only establish a predictive value for m6Avalue for small molecule therapies bioinformatically, but also validate the potentially actionable treatment strategies for targeting aggressive PCa *in vitro* (Fig. 7A-7G).

This study has some limitations. ***First***, our results are mainly derived from bioinformatic analysis, and owing to the retrospective nature of the cohorts, further studies in experimental cohorts are warranted. It is worth noting that our conclusions are confirmed in several independent Oncomine datasets, indicating validity. ***Second***, we stratify the PCa patients into three clusters based on the expression pattern of 24 regulators to represent the distinct global m6A patterns. But, in reality, the precise m6A landscape is unclear due to a lack of large-scale m6A-seq performed in TCGA and Ocomine PCa cohorts. Future effort in dissecting the m6A landscape in a large population will help understand the pathway thoroughly. ***Third***, we interestingly found many differentially spliced events (DSEs) among three m6A_Clusters, accompanying with a group of patients harboring more potential neoantigens detected in C3. However, which splicing events functionally associate with the presentation of neoantigens that contribute to an immune phenotype of TME remains unanswered. ***Fourth***, we have attempted to investigate the relationship between m6Avalue and ADT response in PCa patients using two available datasets (GSE48403 and GSE150368), but failed to reach a conclusion due to a small size of both cohorts (n=7 and 6 patients, respectively) and an intrinsic heterogeneity in PCa. Further examination of this relationship in a large clinical cohort (when feasible) is needed. ***Fifth***, although at population level, our m6Avalue model can efficiently stratify pri-PCa patients and predict their immunotherapeutic response. It is conceivable that this model will have limitation when applying to individual patients or a small group. Essentially, several recent reports ([7-9](#_ENREF_7),[13](#_ENREF_13),[14](#_ENREF_14)) that used similar bioinformatic approaches share the same limitation. However, this limitation may urge an opportunity of combined use of m6Avalue together with other biomarkers to better classify cancer cohort more accurately.

**References:**

1. He, L., Li, H., Wu, A., Peng, Y., Shu, G. and Yin, G. (2019) Functions of N6-methyladenosine and its role in cancer. *Molecular cancer*, **18**, 176.

2. Wang, T., Kong, S., Tao, M. and Ju, S. (2020) The potential role of RNA N6-methyladenosine in Cancer progression. *Molecular cancer*, **19**, 88.

3. Zeng, C., Huang, W., Li, Y. and Weng, H. (2020) Roles of METTL3 in cancer: mechanisms and therapeutic targeting. *Journal of hematology & oncology*, **13**, 117.

4. Zheng, Q.K., Ma, C., Ullah, I., Hu, K., Ma, R.J., Zhang, N. and Sun, Z.G. (2021) Roles of N6-Methyladenosine Demethylase FTO in Malignant Tumors Progression. *OncoTargets and therapy*, **14**, 4837-4846.

5. Lan, N., Lu, Y., Zhang, Y., Pu, S., Xi, H., Nie, X., Liu, J. and Yuan, W. (2020) FTO - A Common Genetic Basis for Obesity and Cancer. *Frontiers in genetics*, **11**, 559138.

6. Li, Y., Xiao, J., Bai, J., Tian, Y., Qu, Y., Chen, X., Wang, Q., Li, X., Zhang, Y. and Xu, J. (2019) Molecular characterization and clinical relevance of m(6)A regulators across 33 cancer types. *Molecular cancer*, **18**, 137.

7. Shen, S., Zhang, R., Jiang, Y., Li, Y., Lin, L., Liu, Z., Zhao, Y., Shen, H., Hu, Z., Wei, Y. *et al.* (2021) Comprehensive analyses of m6A regulators and interactive coding and non-coding RNAs across 32 cancer types. *Molecular cancer*, **20**, 67.

8. Guo, Y., Wang, R., Li, J., Song, Y., Min, J., Zhao, T., Hua, L., Shi, J., Zhang, C., Ma, P. *et al.* (2021) Comprehensive Analysis of m6A RNA Methylation Regulators and the Immune Microenvironment to Aid Immunotherapy in Pancreatic Cancer. *Frontiers in immunology*, **12**, 769425.

9. Zhang, B., Wu, Q., Li, B., Wang, D., Wang, L. and Zhou, Y.L. (2020) m(6)A regulator-mediated methylation modification patterns and tumor microenvironment infiltration characterization in gastric cancer. *Molecular cancer*, **19**, 53.

10. Chong, W., Shang, L., Liu, J., Fang, Z., Du, F., Wu, H., Liu, Y., Wang, Z., Chen, Y., Jia, S. *et al.* (2021) m(6)A regulator-based methylation modification patterns characterized by distinct tumor microenvironment immune profiles in colon cancer. *Theranostics*, **11**, 2201-2217.

11. Bagaev, A., Kotlov, N., Nomie, K., Svekolkin, V., Gafurov, A., Isaeva, O., Osokin, N., Kozlov, I., Frenkel, F., Gancharova, O. *et al.* (2021) Conserved pan-cancer microenvironment subtypes predict response to immunotherapy. *Cancer cell*, **39**, 845-865 e847.

12. Song, L., Cohen, D., Ouyang, Z., Cao, Y., Hu, X. and Liu, X.S. (2021) TRUST4: immune repertoire reconstruction from bulk and single-cell RNA-seq data. *Nat Methods*, **18**, 627-630.

13. Chen, H., Yao, J., Bao, R., Dong, Y., Zhang, T., Du, Y., Wang, G., Ni, D., Xun, Z., Niu, X. *et al.* (2021) Cross-talk of four types of RNA modification writers defines tumor microenvironment and pharmacogenomic landscape in colorectal cancer. *Molecular cancer*, **20**, 29.

14. Zeng, D., Li, M., Zhou, R., Zhang, J., Sun, H., Shi, M., Bin, J., Liao, Y., Rao, J. and Liao, W. (2019) Tumor Microenvironment Characterization in Gastric Cancer Identifies Prognostic and Immunotherapeutically Relevant Gene Signatures. *Cancer immunology research*, **7**, 737-750.

**2. Supplementary Figures**


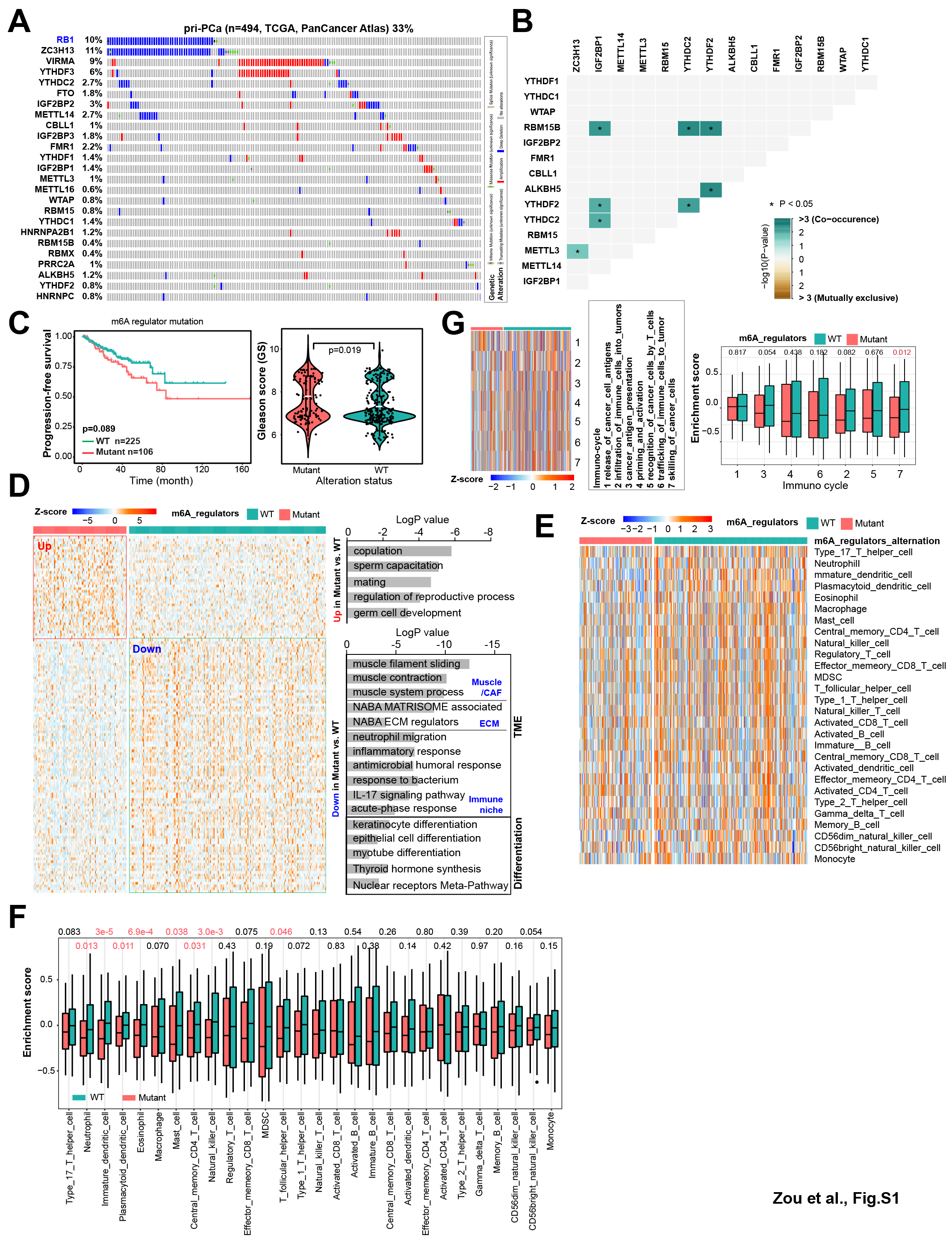


**Figure S1. Genomic alterations of 24 m6A regulators ambiguously classify PCa samples**

**A** The landscape of genomic alterations in 24 m6A regulators in the pan-cancer TCGA-PRAD cohort (n=494). Frequently delated *RB1* (colored in blue) is included as a reference gene. Each bar represents the alteration status of an individual gene for a single patient and the percentage of alterations for each gene in the cohort is provided.

**B** The mutational co-occurrence or exclusion relationship between 24 m6A regulators. Green and golden denote co-occurrence and exclusion, respectively.

**C** Kaplan–Meier plot showing groups with or without genomic mutations in 24 regulators being non-significantly associated with patients’ overall survival (left). Violin plot indicating an association of mutant group with higher Gleason score (GS) (right). Analysis is based on the curated TCGA cohort (n=333) and two samples (TCGA-HC-7740 and TCGA-HC-8265) are omitted due to duplicate existing for each sample in the cohort. Significance were calculated by the long-rank test (left) and Wilcoxon test (right).

**D** Heatmap of differentially expressed genes (DEGs) identified in the mutation versus (vs.) WT group in the curated TCGA cohort (left). Gene ontology (GO) analysis of the 37 upregulated (top) and 95 downregulated genes (bottom) in mutant group by Metascape (right).

**E** and **F** Heatmap presentation (E) and boxplot quantification (F) of the abundance of 28 tumor-infiltrating lymphocytes (TIL) subpopulations calculated by GSVA in mutant vs. WT groups. A small difference in the composition of 28 TIL subsets was noticed. Within the plots, the center lines represent median values, box edges are 75^th^ and 25^th^ percentiles, and dots denote the outliers, respectively. Significance was calculated by the Wilcoxon test.

**G** Cancer-immunity cycle analysis by GSVA between mutation and WT groups. Significance was calculated by the Wilcoxon test.


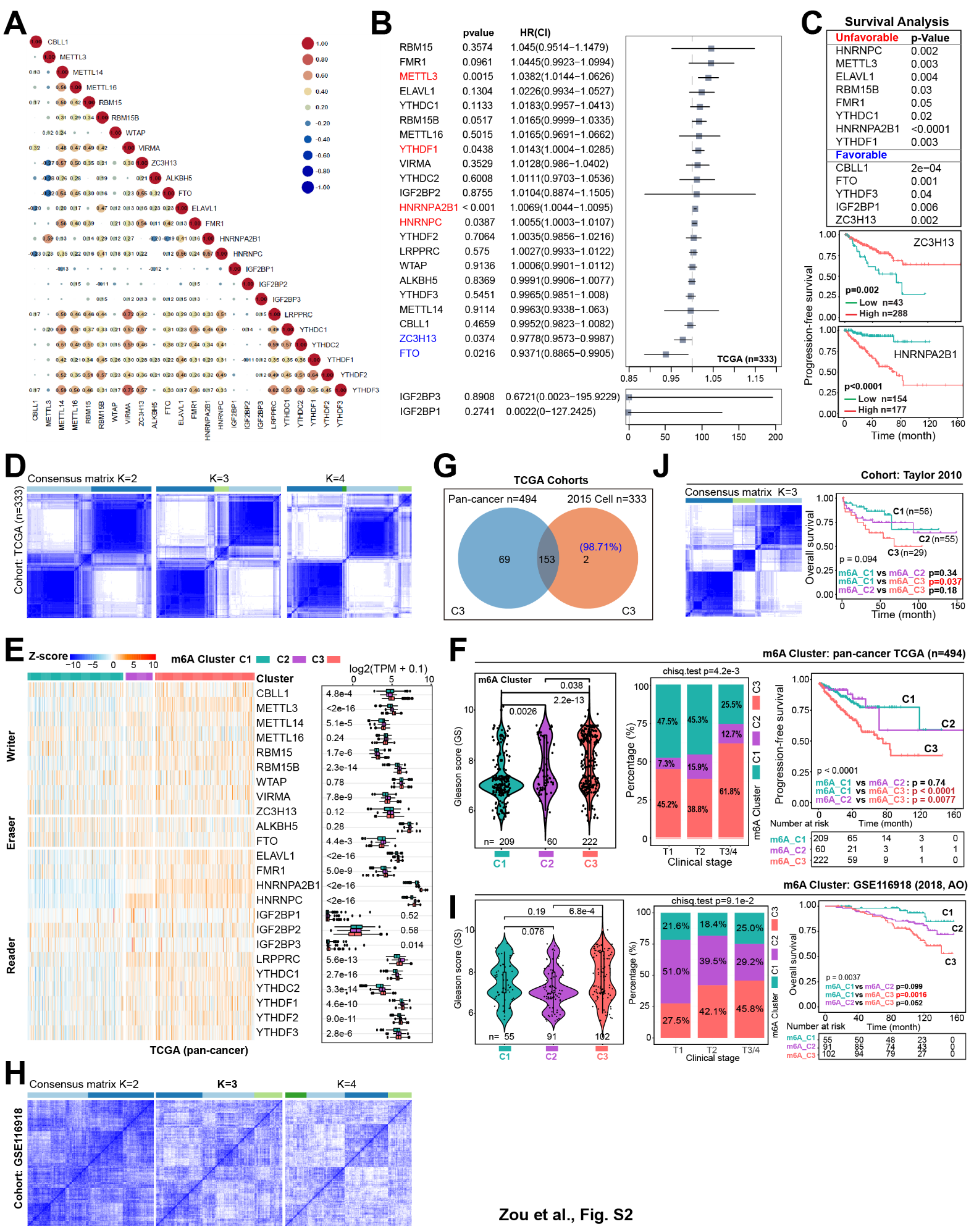


**Figure S2. Distinct m6A modification patterns associate with PCa aggressiveness**

**A** Pearson correlation among the expression of each m6A regulator. Red and blue indicate positive and negative correlation, respectively.

**B** Estimation of prognostic significance for each m6A regulator in the curated TCGA cohort by univariate cox regression. Risk and protective factors are colored in red and blue, respectively.

**C** Survival analysis of individual m6A regulators showing eight unfavorable and 5 favorable genes (upper). Representative unfavorable (HNRNPA2B1) and favorable (ZC3H13) gene plots are displayed (bottom).

**D** Unsupervised clustering based on the expression of 24 m6A regulators showing k=3 as the optimal consensus matrice.

**E-F** Validation in the pan-cancer TCGA cohort (n=494). Heatmap presentation (left) and boxplot quantification (right) of the expression pattern of 24 m6A regulators among three m6A_Clusters. Comparison of GS (F, left), tumor stage (F, middle), and patients’ survival (F, right) showing m6A cluster C1 and C3 as the least and most aggressive PCa subtype, respectively. The proportion of patients clustered into m6A_C2/3 in advanced T3/4 stages (61.8%+12.7%=74.5%) is much higher than that in T1 (45.2%+7.3%=52.5%) and T2 (38.8%+15.9%=54.7%) stages. Notably, four samples are omitted due to a lack of information regarding GS, clinical stage and survival time. Significance was calculated by the Kruskal-Wallis test (E-right, F-left), Chi-square test (F, middle), and long-rank test (F, right).

**G** Patient classification is highly concordant between the curated TCGA (n=333) and pan-cancer TCGA (n=494) PCa cohorts. Almost 99% of patients classified in C3 in the curated TCGA cohort are also classified in C3 in pan-cancer TCGA cohort.

**H** and **I** Validation in independent GSE116918 cohort. Unsupervised clustering of 24 m6A regulators with consensus matrices from K=2 to 4 (H). Comparison of GS (I, left), tumor stage (I, middle), and patients’ survival (I, right) showing m6A_cluster C1 and C3 as the least and most aggressive PCa subtype, respectively. The proportion of patients clustered into C3 in advanced T2 (42.1%) and T3/4 (45.8%) stages is much higher than that in T1 (27.5%) stages. Significance was calculated by the Kruskal-Wallis test (I, left), Chi-square test (I, middle), and long-rank test (I, right).

**J** Validation in another GSE21034 cohort. Unsupervised clustering of expression of 24 m6A regulators in Taylor cohort also showing optimal 3 clusters (left). Kaplan–Meier plot showing an association of C3 with worse survival outcome (right). *p<0.05, **p<0.01, ***p<0.001, and ****p<0.0001.


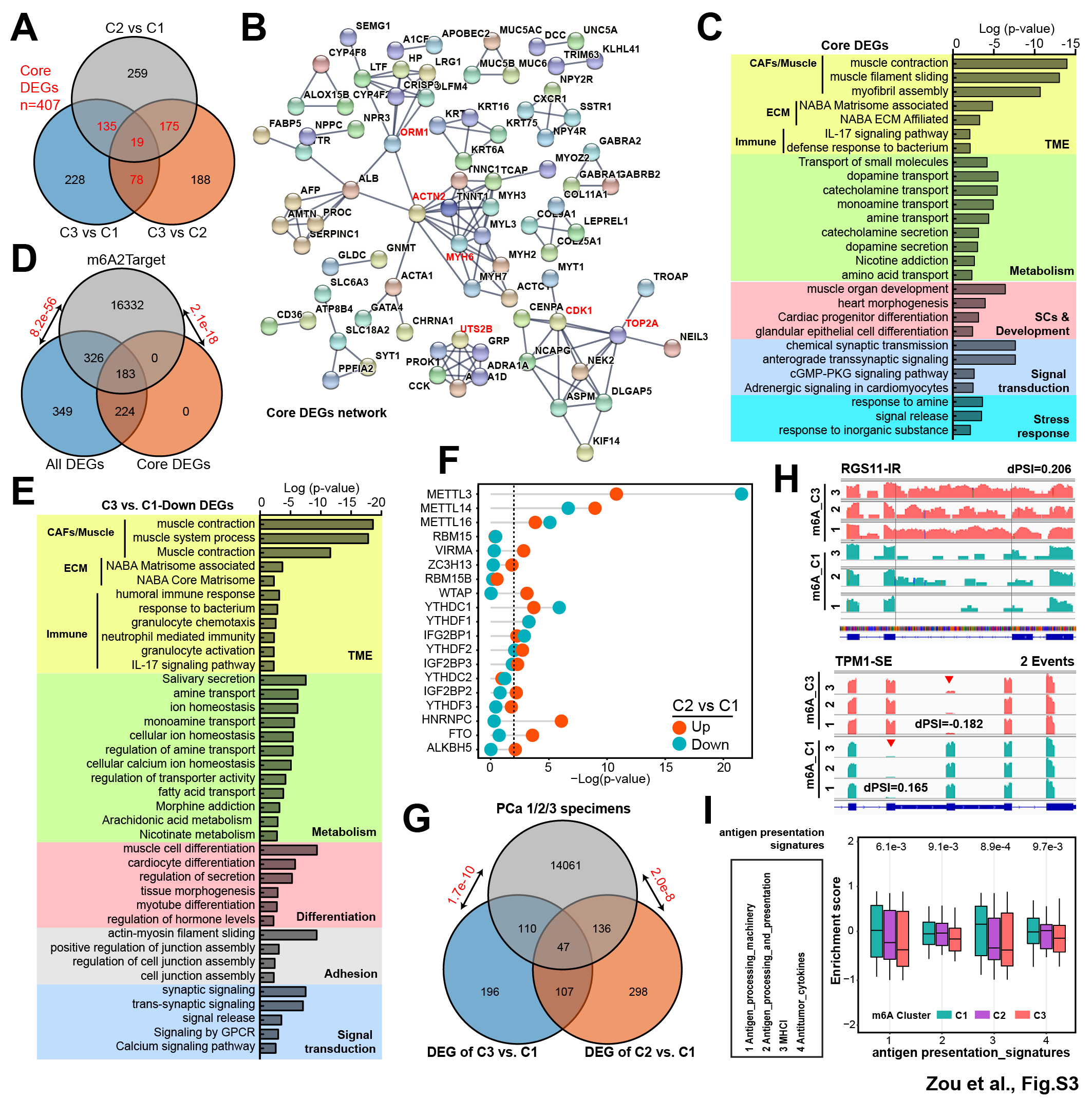


**Figure S3. Distinct m6A modification patterns contribute to TME and splicing dysregulation**

**A** Overlapping of DEGs between any two of three m6A_Clusters identifying 407 core-DEGs as m6A phenotype-associated genes.

**B** Visualization of protein-protein interaction network of 407 core-DEGs by STRING. Several hub genes exhibiting more connections are labeled in red.

**C** GO analysis of 407 core-DEGs by Metascape. Top enriched GO terms were displayed and grouped into five functional categories, with tumor microenvironment (TME) being the top one. The *p*-values were calculated based on the cumulative hypergeometric distribution.

**D** Venn diagram showing overlap of 1082 all DEGs and 407 core-DEGs with potential m6A-target genes reported in m6A2Target database. The *p*-values were analyzed by hypergeometric test.

**E** GO analysis of downregulated genes in C3 vs. C1 by Metascape. Top enriched GO terms were displayed and grouped into five functional categories, with TME being the top one. Overall, the result suggested that tumors in C1 (relatively to C3) are more differentiated, less migrative, and stroma-enriched. The *p*-values were calculated based on the cumulative hypergeometric distribution.

**F** Overlapping of DEGs identified in C2 vs. C1 and potential target genes of distinct m6A regulators reported in m6A2Target database showing subtle differences. Significance was calculated using hypergeometric test.

**G** Venn diagram showing overlap of DEGs identified in C3 vs. C1 and DEGs C2 vs. C1 with m6A-containing genes revealed by our preliminary MeRIP-seq (methylated RNA immunoprecipitation sequencing) in three clinical PCa specimens. The *p*-values were calculated using hypergeometric test.

**H** Examples of splicing dysregulation between C3 and C1. Showing are sashimi plot visualization of IR in RGS11 gene and SE in TPM1 gene in indicated groups. The event ΔPSI (percent of splicing inclusion) values calculated by rMATS were provided. Genomic coordinates were indicated at the bottom.

**I** Boxplot showing the enrichment of four antigen presentation signatures among three m6A_Clusters. Significance was calculated by the Kruskal-Wallis test.


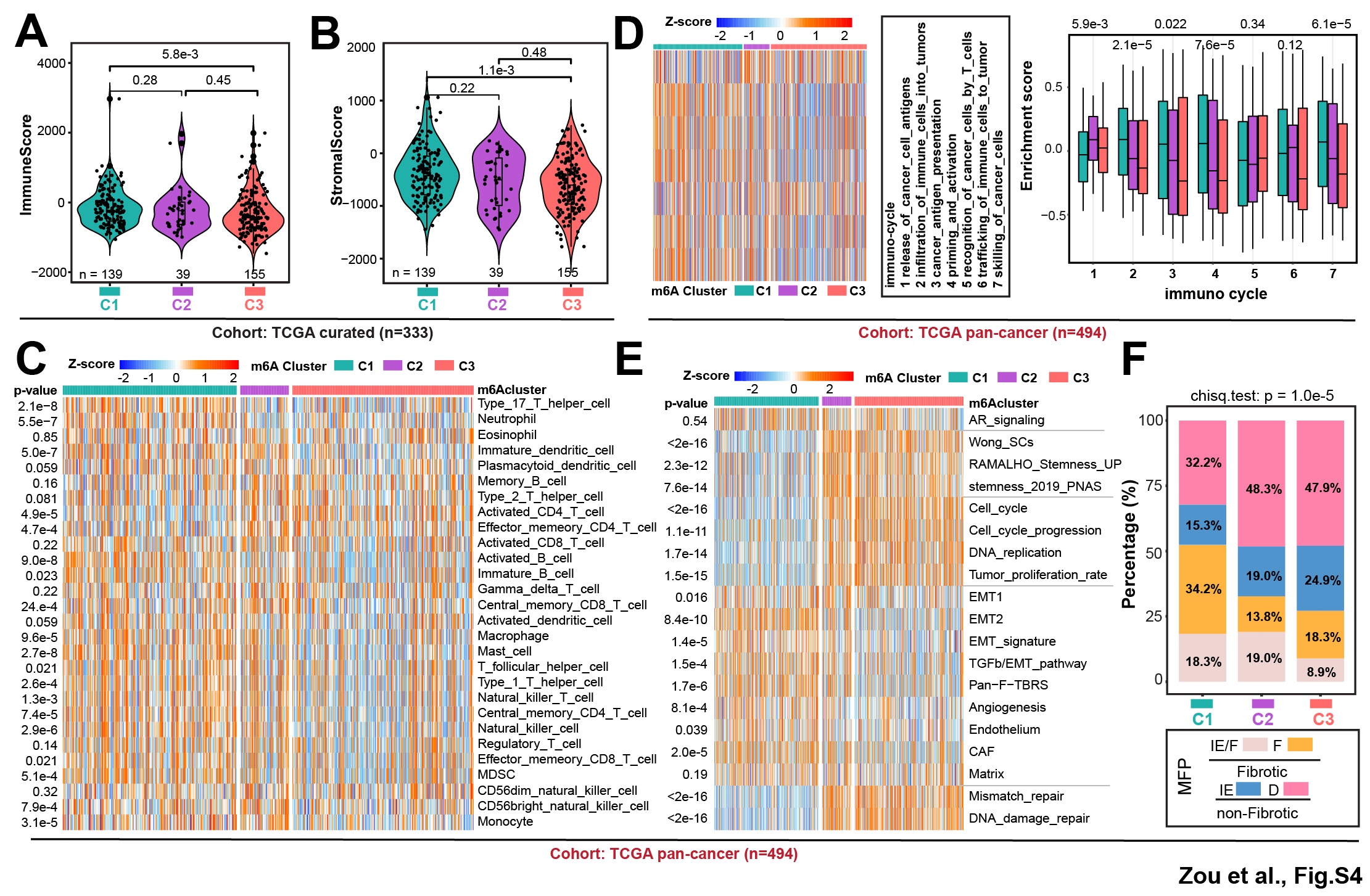


**Figure S4. Validation of cancer hallmarks and immune landscapes among three m6A_Clusters in pan-cancer TCGA cohort**

**A** and **B** Comparison of ImmuneScore (A) and StromalScore (B) in three m6A_Clusters in the curated TCGA cohort. ImmuneScore and StromalScore were calculated by R package ESTIMATE to indicate the overall level of infiltrating immune and stromal cells, respectively.

**C** Heatmap showing enrichment of the 28 TIL subpopulations calculated by GSVA in three m6A_Clusters, with *p*-values labeled on the left. Red and blue in scale bar denote relatively high and low abundance of subpopulations, respectively.

**D** Cancer-immunity cycle analysis by GSVA among three m6A_Clusters.

**E** Heatmap showing enrichment of the indicated gene signatures calculated by GSVA in three m6A_Clusters, with *p*-values labeled on the left.

**F** Distribution of four previously reported TME subtypes in three m6A_Clusters, with p-value (Chi-square test) labeled on top. IE/F: immune-enriched, fibrotic; IE: immune-enriched, non-fibrotic; F: fibrotic; D: immune-depleted. IE/F and F are fibrotic while IE and D are non-fibrotic.

Analyses in C-F were performed based on the pan-cancer TCGA cohort (n=494). Significance was calculated by the Kruskal-Wallis test (A-E).


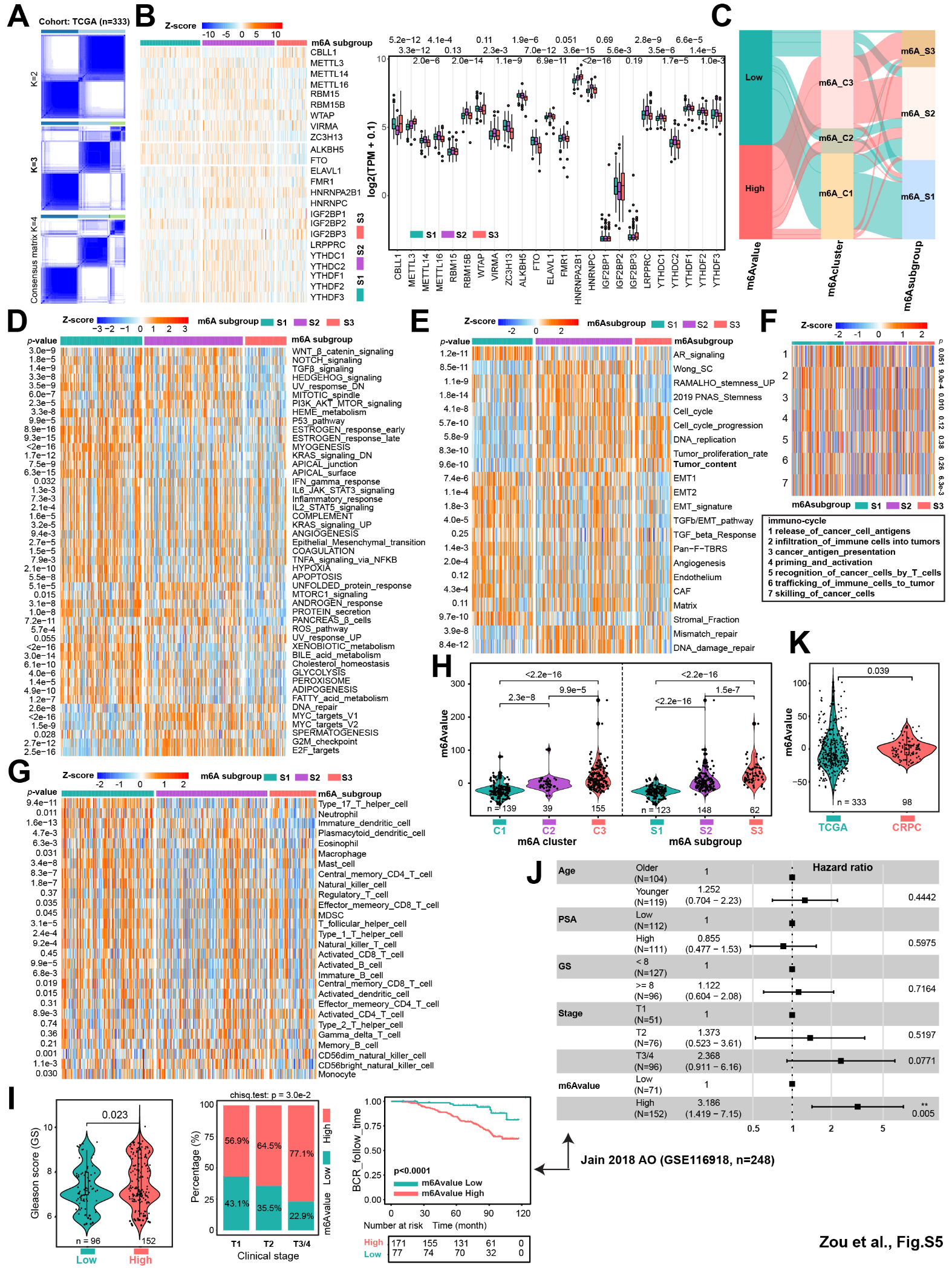


**Figure S5. Three m6A_Subgroups characterize distinct cancer hallmarks and TME phenotypes**

**A** Unsupervised clustering of 407 core-DEGs showing k=3 as the optimal consensus matrix, with a clear separation of the curated TCGA cohort into three subgroups (m6A_Subgroup1/2/3).

**B** Heatmap (left) and boxplot (right) showing differences in expression of the 24 m6A regulators among three m6A_Subgroups.

**C** Alluvial diagram showing the compositional changes of m6Avalue groups, m6A_Clusters and m6A_Subgroups.

**D** Heatmap showing enrichment of the representative cancer hallmarks from MSigDB by GSVA in three m6A_Subgroups in the curated TCGA cohort, with p-values labeled on the left.

**E** Pathway analysis of different m6A modification patterns. Heatmap showing enrichment of the indicated signatures among three m6A_Subgroups.

**F** Cancer-immunity cycle analysis by GSVA showing S1 and S3 as the subgroup with the highest and lowest anti-cancer immunity, respectively.

**G** Heatmap showing enrichment of the 28 TIL subpopulations calculated by GSVA in three m6A_Subgroups, with *p*-values labeled on the left.

**H** Violin plot showing the m6Avalues for tumors classified in either m6A_Clusters or m6A_Subgroups.

**I** and **J** Validation of m6Avalue model in independent GSE116918 cohort. Comparison of GS (I, left), tumor stage (I, middle, Chi-square test), and patient survival outcome (I, right, long-rank test) showing m6Avalue-high group as the aggressive one. Patients’ tumors with higher clinical stages tend to have higher m6Avalues (70.8% in T3/4, 53.9% in T2, and 49.0% in T1). Multivariate Cox regression analysis showing m6Avalue as an independent prognostic factor among other indicated clinical parameters (J).

**K** CRPC samples have higher m6Avalue compared with pri-PCa.

Analyses in A-H were based on the curated TCGA cohort. Unless otherwise stated, significance was calculated by the Kruskal-Wallis test (B, D, E, F, G, H) and Wilcoxon test (K, I-left), respectively.


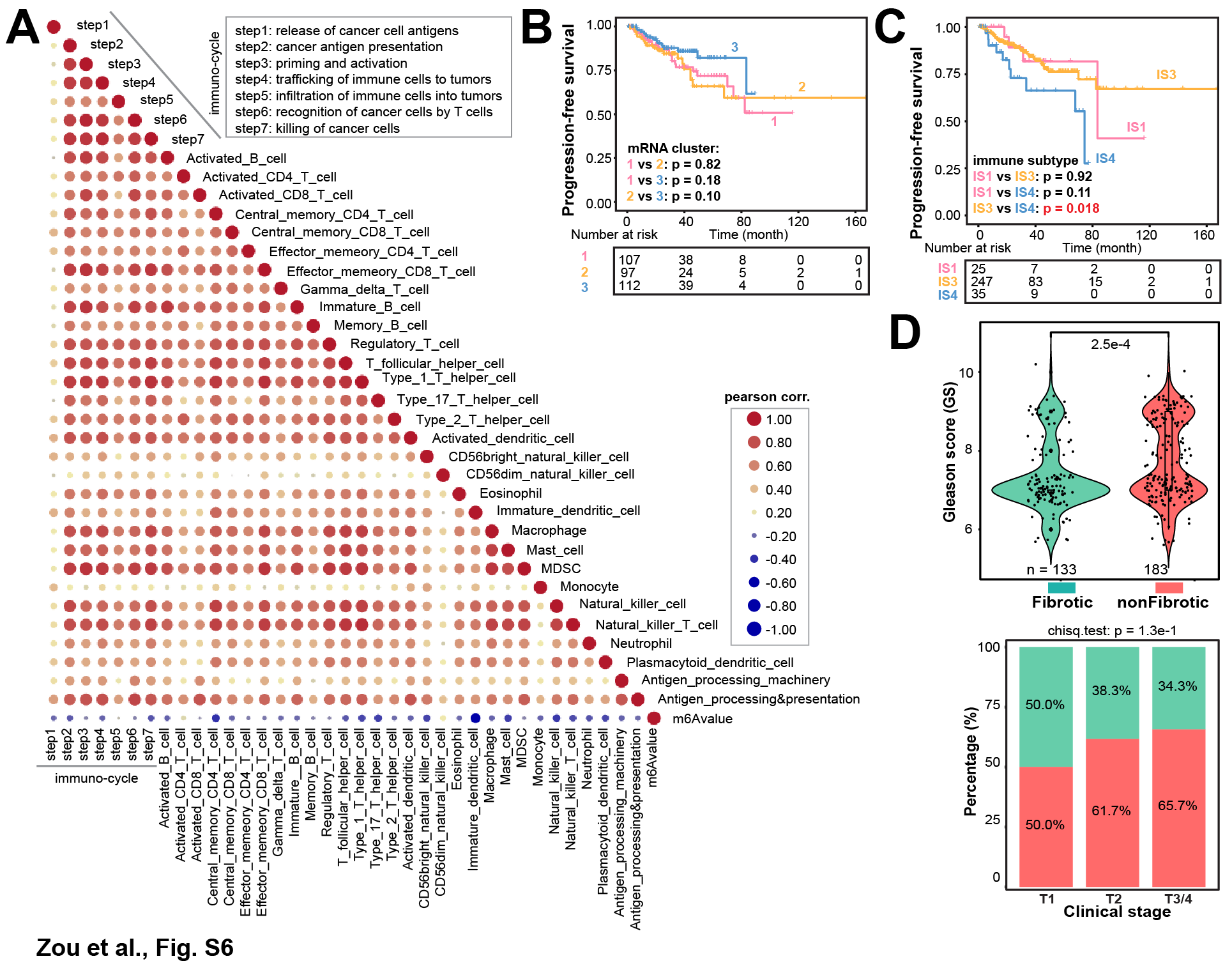


**Figure S6. The m6Avalue negatively correlates with TME immune features**

**A** Pearson correlation of m6Avalue with cancer immune-cycles and abundance of 28 TIL subpopulations. Positive and negative correlation were colored in red and blue, respectively.

**B** and **C** Kaplan–Meier analysis showing survival outcomes of patients classified in different mRNA_clusters (B) and Immune_Subtypes (C). The *p*-values were calculated using long-rank test.

**D** Violin plot showing m6Avalue of tumors with fibrotic or nonfibrotic phenotype (Wilcoxon test, upper). Prostate tumors with higher clinical stage tend to exhibit nonfibrotic phenotype (Chi-square test, bottom).


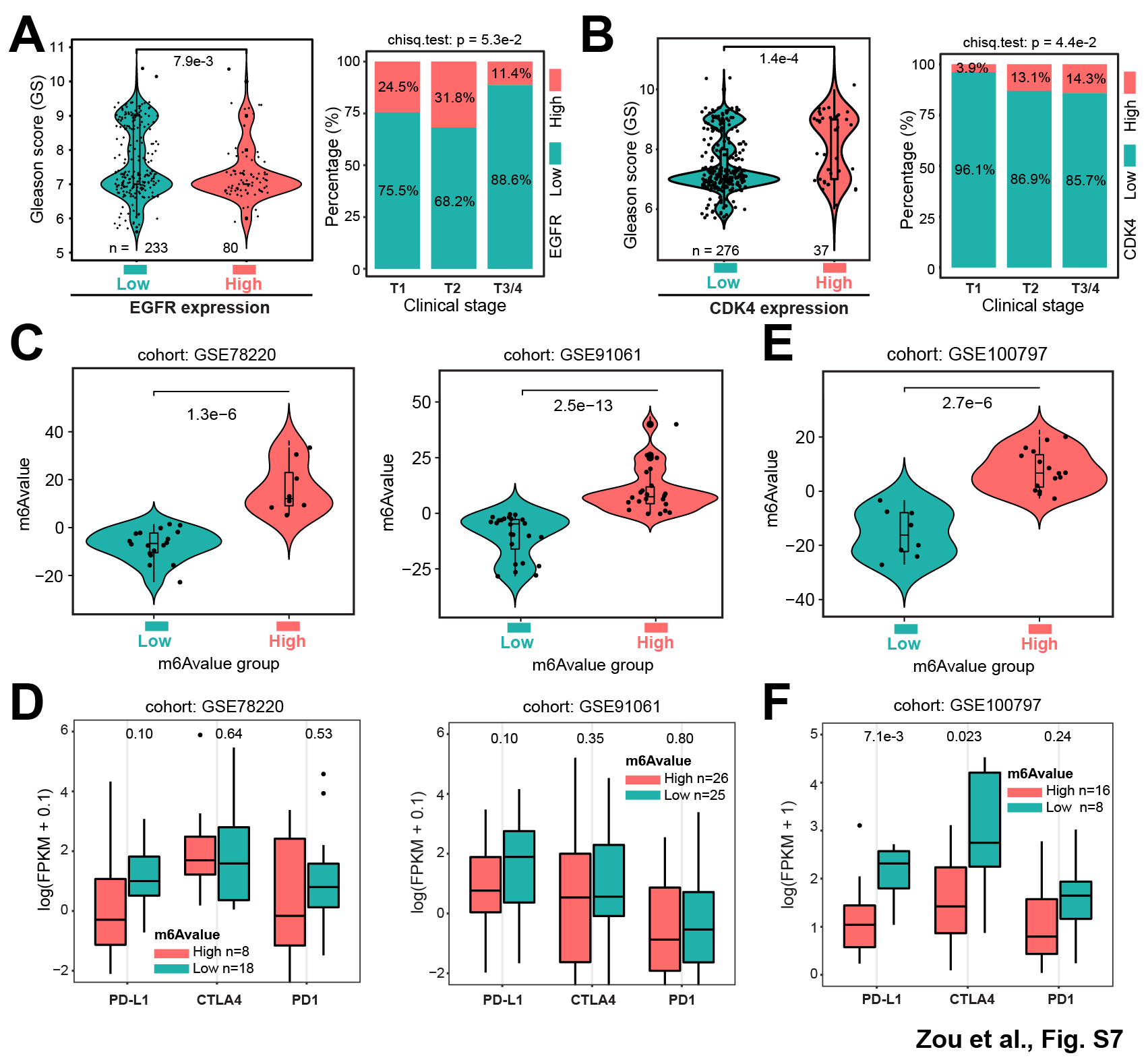


**Figure S7. m6A modification patterns stratify patients with distinct outcomes after treatments**

**A** Comparison of GS (left) and tumor stage (right) indicates that prostate tumors highly expressing EGFR are relatively indolent than their counterparts.

**B** Comparison of GS (left) and tumor stage (right) indicates that prostate tumors highly expressing CDK4 are relatively aggressive than their counterparts.

**C** and **E** Comparison of m6Avalue between its high and low groups in the indicated cohorts.

**D** and **F** Boxplot showing expression of the immune checkpoints (PD-1, PD-L1 and CTLA4) between m6Avalue high and low groups in the indicated cohorts.

Significance for comparison of two groups in A-F and for comparison of three columns in A-B was calculated by the Wilcoxon test and Chi-square test, respectively.

**3. Supplementary Tables**

**Table S1: List of potential 14,354 m6A-modified genes identified in prostate tissues.**

**Table S2: Summary of gene signatures used in this study.**

**Table S3: Master matrix integrating multi-layers of information.**

This table integrates clinical information, classification (m6A_Cluster, m6A_Subgroup and m6Avalue) and enrichment score of selected signatures (including 50 cancer hallmarks, 7 immune cycle steps, 28 TIL infiltration and other 35 curated signatures).

**Table S4: List of DEGs identified in four comparisons.**

**Table S5: Overlap of DEGs among m6A_Clusters with potential** **target genes of m6A regulators.**
